# Life Cycle Complexity Shapes Parasite Sharing Amongst Migratory and Resident Hosts

**DOI:** 10.1111/mec.70657

**Published:** 2026-11-10

**Authors:** Sarah Nichols, Andrea Estandía, Catherine M. Young, Jaslyn Allnutt, Jonathan T. Coleman, Judith Little, Gregory Little, Sonya M. Clegg, Beth Okamura

**Affiliations:** ^1^ Department of Biology University of Oxford Oxford UK; ^2^ Life Sciences, Natural History Museum London UK; ^3^ Fenner School of Environment and Society Australian National University Canberra Australian Capital Territory Australia; ^4^ Institute for Marine and Antarctic Studies University of Tasmania Hobart Tasmania Australia; ^5^ Shailer Park Queensland Australia; ^6^ Boolaroo New South Wales Australia; ^7^ School of Environment and Science Griffith University Nathan Queensland Australia

**Keywords:** host–parasite network, migration, parasite dispersal, parasite life histories, transmission

## Abstract

Migratory animals are potentially important parasite dispersers. However, understanding the impact of host migration on parasite distributions is challenging because variation in parasite life histories can mediate dispersal potential. Furthermore, studies of parasite dispersal via host migration often compare parasites in different species of migrant and resident hosts, despite differences in host phylogeny, ecology, and exposure history, which can complicate ascertaining the impact of migration. The Tasmanian population of silvereye *Zosterops lateralis lateralis* ) exhibits partial migration. Some members of the population overwinter on the Australian mainland, where they co‐occur with residents of another silvereye subspecies (*Z. l. cornwalli*). Utilising targeted PCR, metabarcoding and high‐throughput sequencing, we characterise a range of parasite genera from blood and faecal samples in Tasmanian resident, migrant and mainland resident birds. Greater parasite richness was documented in migrant individuals, possibly reflecting exposure to varied parasite faunas at stopovers or during overwintering. The increased richness was primarily driven by parasites with simple life cycles. Reduced sharing of parasites with complex life cycles amongst migratory and resident host populations may be explained by ecological constraints in establishing transmission, such as vector or secondary host availability. Our study exemplifies the utility of molecular methods for characterising multi‐parasite systems and makes use of a single host species to provide insights on patterns of parasite distributions that result from migratory movements. Knowing how parasite life histories constrain or promote dispersal via regular host migration will be essential for predicting future patterns of pathogen dispersal under climate change and shifting migratory regimes.

## 1 Introduction

Migratory animals embarking on seasonal long‐distance movements typically encounter a variety of habitats and organisms, exposing them to a wide array of pathogens and parasites. Hosts may consequently lose, swap, or gain parasites as a result of their seasonal migratory movements (Clay 2003; Clark et al. 2015). Such migration‐related exposure to parasites can profoundly impact the dynamics of infectious disease (Altizer et al. 2011). Yet, the impact of migration on parasite dispersal and distribution remains difficult to predict, particularly because processes that cause migratory hosts to gain and lose parasites may be happening concurrently, and because similar patterns of parasite distributions can arise via different mechanisms (Peacock et al. 2020). With global climate change altering the timing, routes, and scale of animal migrations (Malcolm et al. 2002; Mills 2005; Bussière et al. 2015; Kristensen et al. 2015), elucidating the impact of host migration on parasite distribution has become increasingly urgent (Garamszegi 2011; Fritzsche Mckay and Hoye 2016).

Several hypotheses have been established regarding dynamics of parasites in migratory hosts. Hosts may gain a diverse parasite community via exposure to multiple parasite assemblages during stopovers or upon arrival, i.e., “migratory exposure” (Koprivnikar and Leung 2015; Leung and Koprivnikar 2016). Parasites may also alter migratory host behaviours by imposing energetic costs which lead to trade‐offs amongst functions (DeGroote and Rodewald 2010). For example, physiological trade‐offs with the immune system (Eikenaar and Hegemann 2016), may result in less variation at the immune loci (O'Connor et al. 2018), and consequently higher parasite loads in migratory animals (Altizer et al. 2011). Contrastingly, migrants have also been hypothesised to have evolved varied immune loci as a response to frequent parasite exposure (Hegemann et al. 2015), resulting in them having a less diverse parasite community. Alternatively, the energetic cost of migration may lead to behavioural trade‐offs. Thus, the “migratory escape” hypothesis proposes that periodic migration enables hosts to avoid the accumulation of parasites (Kendzel et al. 2023). The detrimental impact of infection on host fitness may also lead to loss of parasites from host populations via “migratory culling” (Bartel et al. 2011; Slowinski et al. 2018). Such evolutionary responses from hosts may, in turn, lead to changes in parasite phenology (Ishtiaq and Renner 2020), transmission, or survival strategies (Poulin and de Angeli Dutra 2021). Disentangling the effects of these non‐mutually exclusive hypotheses is challenging, particularly as they may operate in tandem with different parasite genera imposing opposing effects within the same host (Gutiérrez et al. 2019).

Empirical studies that compare parasite diversity in migratory and resident birds have produced varied results. Greater parasite richness in migratory compared to resident host species has been documented for nematodes (Koprivnikar and Leung 2015; Leung and Koprivnikar 2016); whereas for haemosporidians, the causative agents of avian malaria, results are more varied. Some studies have found higher richness in migratory bird species (de Angeli Dutra, Fecchio, et al. 2021) while others have found no detectable difference between resident and migrant species (Ricklefs et al. 2017). Similarly, results often seem to vary by parasite genus. Perrin et al. (2025) recorded higher *Plasmodium* richness in migrants than in residents but found no difference for *Haemoproteus*. La Chapelle et al. (2023) reported greater *Haemoproteus* diversity in migrants than in residents restricted to a single continent. We are not aware of studies documenting consistently lower haemosporidian richness in migrant hosts.

Quantifying parasite diversity in migratory hosts is important for understanding the potential for migration‐facilitated parasite sharing. In terms of sharing at the host species community level, studies have yielded mixed results. Perrin et al. (2025) compared 697 bird species and found that migratory species shared many *Haemoproteus* and *Plasmodium* lineages with resident species encountered along the African‐Eurasian and Americas flyways, implying that migrants may disperse and/or acquire parasites during their journeys. Another study, based on 896 bird species sampled across South America, revealed that communities with a higher proportion of migrant birds supported higher haemosporidian lineage richness (de Angeli Dutra, Filion, et al. 2021). In contrast, other studies have found that various species of co‐occurring migratory and resident Passeriformes harbour largely distinct haemosporidian communities at stopover and overwintering sites in South and North America (Pulgarín‐R et al. 2019; Soares et al. 2020; Galen et al. 2024). Such results may reflect differences in vector availability or competency across the migrant hosts' annual cycle. For example, Galen et al. (2024) examined haemosporidian parasites in 63 species of birds at a stopover site in Pennsylvania (United States) and found that migratory birds shared a core set of haemosporidians with residents. However, migratory bird species also carried several unique haemosporidian lineages associated with different biogeographic regions that were characterised by different vector communities. This suggests that vector ecology may preclude transmission for some haemosporidian parasites. Such results highlight that dispersal of parasites with complex life cycles may be constrained by the availability of secondary hosts or vectors. Therefore, it is important to assess parasites with differing life cycles when considering the impact of migration on parasite sharing.

To date, studies that attempt to quantify parasite diversity and subsequent sharing between resident and migrant hosts often rely on species‐level comparisons. Such comparisons do not necessarily account for differences in host phylogeny, ecology, and exposure history, which may confound efforts to resolve the effects of migration on parasite communities (Binning et al. 2022). Partial migrant systems, in which individual‐level variation in migratory behaviour occurs, provide a valuable opportunity to understand parasite transmission dynamics during migration in a single host species. Although partial migration is relatively common in birds (Lundberg 1988; Chapman et al. 2011), distinguishing migrants from non‐migrants without individual tracking can be challenging. However, this issue can be avoided by strategically sampling during the migratory period, when migrant and resident birds self‐sort. Furthermore, systems in which migrating individuals encounter another morphologically distinct subspecies during the migratory period enable further resident and migrant comparisons to be made. Such systems offer a tractable framework for studying parasite sharing between resident and migratory populations.

The Tasmanian population of silvereye ( *Zosterops lateralis lateralis* ), a passerine bird of Australia and the southwest Pacific, undergoes partial migration (Chan and Kikkawa 1997). During the winter, some individuals remain on the Tasmanian breeding grounds while others cross the Bass Strait to overwinter on the Australian mainland, where they co‐occur with year‐round residents of another morphologically distinct subspecies of silvereye ( *Zosterops lateralis cornwalli* ). We take advantage of this system of resident and partial migrant silvereye subspecies to understand how migration shapes parasite communities. First, because Tasmanian migrants and Tasmanian residents belong to the same subspecies (*Z. l. lateralis*), differences in their parasite communities can be more readily attributed to migratory behaviour rather than differing ecological and evolutionary histories reflecting subspecies level differences. Secondly, sampling during the migratory period, when the Tasmanian migrants and residents are spatially separated by the Bass Strait, allows individuals to be assigned to migrant or resident categories without individual tracking. Third, the morphologically distinct mainland resident subspecies (*Z. l. cornwalli*) with which overwintering migrants co‐occur, provides a second resident comparison: migrants can be compared to both residents they leave behind and join. Comparing these three groups allows the direction of parasite sharing to be inferred, which is not possible when migrants and residents are sampled at a single point in the annual cycle. By utilising high‐throughput sequencing (HTS) methods we characterise a broad range of parasite genera with varied life histories detected in host faeces and blood. This enables us to directly address whether differences in parasite richness between resident and migrant birds depend on life cycle complexity, and if some parasites are more broadly shared between host groups than others.

## 2 Materials and Methods

### 2.1 Sample Collection

We conducted sampling from May to July 2022 when the Tasmanian population of silvereye (*Z. l. lateralis*) undergoes partial migration (Chan and Kikkawa 1997). During May and June, samples from overwintering migratory individuals were collected in Queensland (QLD) and New South Wales (NSW) on the Australian mainland. We also collected samples from individuals of the year‐round resident subspecies, *Z. l. cornwalli*, which were present in the same area as migrants. Individuals of the two subspecies were discriminated by plumage differences (Little et al. 2020). Samples from individual Tasmanian residents were collected during June 2022. Figure 1A illustrates the sampling locations and partial migrant route.

**FIGURE 1:**
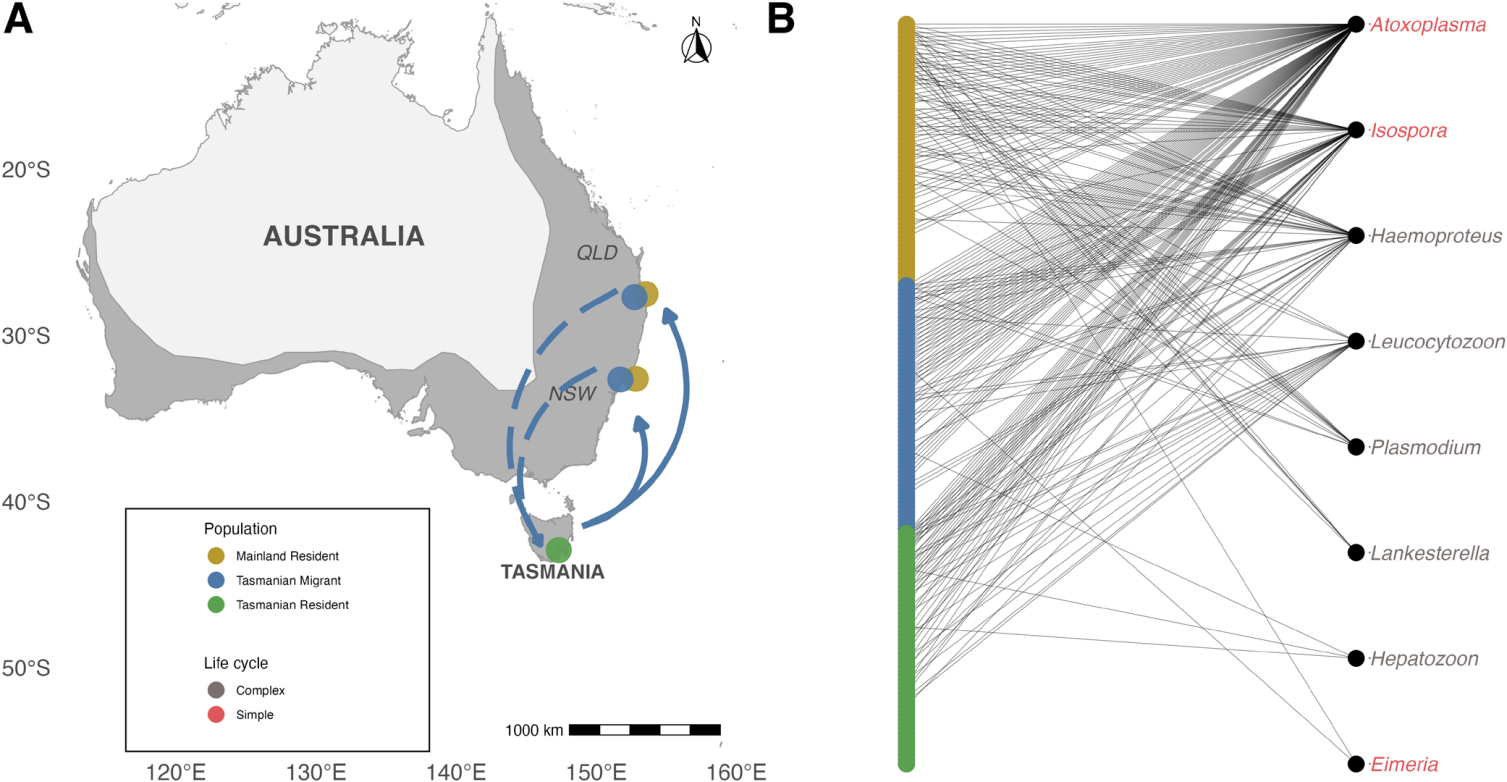
(A) Map showing silvereye distribution in Australia (dark grey) and sampling locations (indicated by circles) for Tasmanian residents (green, *n* = 53), Tasmanian migrants (blue, total *n* = 56, comprising New South Wales [NSW] = 41 and Queensland [QLD] = 15) and mainland residents (yellow, total *n* = 59, comprising NSW = 43 and QLD = 16), the blue arrows indicate the north–south migrant movement, Tasmanian migrants were sampled after they arrived on the mainland (direction of travel indicated by solid blue line), before their presumed return to Tasmania (dashed line). (B) Corresponding bipartite network constructed for 8 parasite genera and 168 silvereye individuals from the three silvereye populations. Text colour indicates simple (red) or complex (grey) life cycle.

Faecal samples were collected according to a protocol adapted from Knutie and Gotanda (2018). Birds were captured using a mist net and subsequently placed in a ventilated, flat‐bottomed paper bag lined with two sterile weigh boats covered by wire mesh that acted as a perch (Figure S1). Individual birds were left in the bag for approximately 3 min to defecate. Faecal samples were transferred to 1.5 mL microfuge tubes containing 1 mL 100% ethanol. A blood sample was taken by pricking the brachial vein with a sterile needle and collecting the resulting blood droplet in a glass capillary tube. Capillary tubes were snapped to size then placed in 1.5 mL microfuge tubes with Queen's lysis buffer (0.01 M Tris, 0.01 M NaCl, 0.01 M EDTA and 0.03 M n‐lauroylsarcosine [10% w/v], pH 7.5, modified from Seutin et al. 1991). All samples were refrigerated until they could be frozen at −20°C. Birds were fitted with an individually numbered band provided by the Australian Bird and Bat Banding Scheme. A suite of morphometrics were measured with dial callipers ±0.1 mm (tarsus length as well as bill length, depth and width) and a flat rule ±1 mm (wing length and tail length). Weight was measured with a Pesola scale. Birds were then released.

### 2.2 DNA Extractions

Faecal DNA extractions were carried out using the Qiagen DNeasy PowerSoil Pro Kit (Qiagen, Germany) according to the manufacturer's instructions. Blood extractions were conducted using a modified phenol‐chloroform protocol (Sambrook and Russell 2006). Samples were incubated overnight at 55°C with rotation, adding approximately 100 μL of blood to 250 μL DIGSOL extraction buffer (0.02 M EDTA, 0.05 M Tris–HCl [pH 8.0], 0.4 M NaCl, 0.5% SDS) and 10 μL Proteinase K (20 mg/mL). Digested samples were added to 250 μL phenol:chloroform:isoamyl alcohol (25:24:1) and rotated for 10 min followed by centrifugation at 10,000 rpm for 10 min. The aqueous phase was transferred to a new microfuge tube; rotation and centrifugation were repeated with the addition of 250 μL phenol:chloroform:isoamyl alcohol (25:24:1). This step was then repeated with the addition of 250 μL chloroform:isolamyl alcohol (24:1); and 2 volumes of cold 100% ethanol, 1 volume of 2.5 M ammonium acetate and 1 μL glycogen to precipitate the DNA. Samples were stored at −20°C for at least 12 h before further centrifugation for 10 min at 15,000 rpm at 4°C, the precipitate was washed with 500 μL cold 70% ethanol. The process of centrifugation and washing was done twice more. The precipitated DNA was left to dry at room temperature before resuspension in 25 μL of TrisEDTA (TE) buffer (0.01 M Tris–HCL [pH 8.0], 0.0001 M EDTA). Each batch of 24 samples included one negative extraction control.

### 2.3 Sexing

Silvereyes are sexually monomorphic (Wilson and Kikkawa 1988), therefore, individuals were sexed genetically. We used two sets of markers: P2D‐P8 (Dawson et al. 2012) and Z002B (Dawson 2007). PCR amplification was conducted in a total volume of 2 μL, consisting of 1 μL QIAGEN Multiplex PCR mix (5X, QIAGEN Inc., Germany) and 1 μL of primer mix, containing 5 μM of each primer, ddH_2_O and ~15 ng of DNA, obtained by allowing 1 μL of resuspended DNA extract to evaporate. The PCR conditions involved an initial denaturation stage at 95°C for 15 min, followed by amplification for 35 cycles (94°C for 30 s, 48°C [P2D‐P8] or 56°C [Z002B] for 90 s and 72°C for 60 s, and a final extension for 30 min at 60°C). PCR products were diluted in ddH_2_O to a ratio of 1:2500–1:5000 and analysed on an ABI 3730 48‐capillary DNA Analyser using formamide and GeneScan‐500 ROX size‐standard (Applied Biosystems, Warrington, UK). Alleles were scored using GENEMAPPER v5 software (Applied Biosystems, California, United States).

### 2.4 18S Anti‐Metazoan Metabarcoding

A set of 18S anti‐metazoan primers, 3NDF (Cavalier‐Smith et al. 2009) and the “universal non‐metazoan” reverse primer, 18s‐EUK1134‐R (Bower et al. 2004), were used to amplify the V4 region of the 18S SSU rDNA. We chose these primers because the 18S region is highly conserved across taxa, but shows substantial variation amongst taxonomic groups (van de Peer et al. 1997; Wuyts et al. 2000; Stoeck et al. 2010). Furthermore, this region is frequently used to characterise parasites (Kounosu et al. 2019), providing comparatively good representation in reference databases compared to other regions. The primers were designed to avoid amplifying metazoans as otherwise the resultant sequencing reads would likely be dominated by the host. The primers were modified to include annealing sites for indexing. The primer sequences were as follows:

Forward primer: 5′‐ACACTCTTTCCCTACACGACGCTCTTCCGATCTNNNNNGCAAGTCTGGTGCCAG‐3′.

Reverse primer: 5′‐GTGACTGGAGTTCAGACGTGTGCTCTTCCGATCTCTTTAARTTTCASYCTTGCG‐3′.

PCR1 reactions had a final volume of 20 μL and included Qiagen 5X Multiplex PCR Master Mix (Qiagen, Germany), 2 μL of primer at 5 μM (final conc. 0.5 M), 1 μL DNA (ca. 10 ng/μl) and sterile ddH2O. The thermal cycling conditions were as follows: 95°C for 15 min, 35 cycles of 95°C for 30 s, 53°C for 45 s and 72°C for 1 min with a final extension at 72°C for 10 min. Two negative controls were included for every 30 amplifications: a PCR control (using 1 μL ddH_2_O instead of DNA) and an extraction control (using the negative controls generated during DNA extractions). PCR reactions produced a ~540 bp product.

### 2.5 Library Preparation and Sequencing

Successfully amplified samples were cleaned with Promega ProNex Magnetic Beads using a ratio of 1.15X (v/v) beads to product, according to the manufacturer's instructions (Promega Inc., United States). Each sample was dual indexed using a PCR reaction that included 5X Meridian Bioscience MyTaq HS Mix, Fi5 and Fi7 primers (at 10 μM), sterile ddH_2_O and product from PCR1 in a total volume of 20 μL. The PCR conditions were 95°C for 15 min; 8 cycles of 98°C for 10 s, 65°C for 30 s and 72°C for 30 s and a final extension at 72°C for 5 min.

We used the Agilent TapeStation (Agilent Technologies Inc., United States) to confirm the index primers had successfully ligated and subsequently conducted a second clean‐up using a 1X (v/v) ratio of Promega ProNex Magnetic Beads. The concentration of cleaned product (ng/μl) was quantified using the Promega QuantiFluor. Pools were created by combining equal ratios of amplicons from each sample, which were further quantified by qPCR. A 100–1000‐ and 10,000‐fold dilution was prepared for each pool with sample dilution buffer (10 mM Tris, pH 8.0 and 0.05% Tween 20X) and SYBR FAST mix and primers. The thermal cycling conditions were 5 min at 95°C, 35 cycles of 30 s at 95°C and 45 s at 60°C. Pools were further amalgamated and quantified by qPCR until a single pool remained. A High Sensitivity D1000 ScreenTape was used to ascertain the size and quantity of the final product using an Agilent TapeStation.

The final pool was sequenced on a PacBio Sequel II at the Natural Environment Research Council Environmental Omics Facility, University of Liverpool, UK. Library loading was achieved by diffusion, with all the samples loaded onto one SMRT cell 8 M, using the Sequel II Primer V3.1.1.5, Sequel II Binding Kit V3.1. PacBio CCS reads (mean passes = 20, median read quality = Q37) were demultiplexed and Illumina primers were removed using the cutadapt software (Martin 2011).

### 2.6 Calling ASVs and Taxonomic Assignment

The *DADA2* package (Callahan et al. 2016) was used to clean and call Amplicon Sequence Variants (ASVs) from the data. Reads with a minimum quality score of Q30 and those within the minimum (475 bp) and maximum (675 bp) lengths were retained. Following filtering, reads were dereplicated. Dereplicating reduces computation time by amalgamating unique sequences while retaining information about their abundance in the dataset. The samples were subsequently denoised using the learned error rates to remove likely sequencing errors. Chimeras were also removed.

Taxonomic identification of metabarcoding data is often based on sequence similarity. However, sequence similarity ignores evolutionary context (Czech et al. 2022) and is known to give rise to erroneous identifications (Koski and Golding 2001), particularly when there are limited reference sequences available (Barbera et al. 2018). For these reasons, we utilised phylogenetic placement to taxonomically assign ASVs. Phylogenetic placement requires a reference tree which represents sequences spanning the genetic diversity likely to be present in a sample (Czech et al. 2022). We used general 18S anti‐metazoan primers; therefore, we needed to include a range of eukaryotic sequences. To do this, we utilised the 211 sequences curated by Rapp and Wolf (2024) spanning the eukaryotic tree of life, supplemented with additional parasitic sequences that we have previously identified in silvereyes (Nichols et al. 2026). A full list of accession numbers used to build the tree is available in Table S1. We trimmed the reference sequences to encompass the region amplified by our primers using cutadapt (Martin 2011) and subsequently removed any sequences that exceeded 1000 bp as these were assumed to be erroneous (expected amplicon size is 600 bp). Next, we created a multiple sequence alignment (MSA) using MAFFT (Katoh and Standley 2013) and trimmed aligned sequences using trimAL (Capella‐Gutiérrez et al. 2009), removing any alignment columns where more than 50% of sequences had gaps. We used ModelTest‐NG to select a substitution model (GTR + G4; Darriba et al. 2020) and inferred a phylogenetic tree using RAxML‐NG (referred to as a maximum likelihood [ML] tree) (Kozlov et al. 2019). The alignment was reduced for identical sequences, and a ML tree search was conducted with 25 parsimony and 25 random starting trees. Felsenstein bootstrap proportion (FBP) was calculated from 1000 bootstrap replicates to assess node support (Felsenstein 1985). We did not root the tree as this is not required for phylogenetic placement (Czech et al. 2022). Next, we aligned ASVs to the reference MSA using PaPaRa (Berger and Stamatakis 2012), placed them onto the reference tree using EPA‐ng (Barbera et al. 2018) and finally, converted placements to taxonomic assignments with gappa (Czech et al. 2020). Taxonomic assignments with a Likelihood Weight Ratio (LWR) of 0.7 were retained for further analysis.

After taxonomic assignment, we used the *microDecon* package to identify and remove potential contaminants, using the 26 sequenced negative controls generated during the PCR and extraction steps as references (Mcknight et al. 2019). To account for variation in sequencing depth amongst samples, we standardised read depth by rarefying each sample to a minimum sample coverage of 0.9, following the approach recommended by Chiu and Chao (2016).

We searched the available literature to compile life history information for each of the identified parasite genera (Table S2). Specifically, we collated information on previously recorded primary hosts according to the following categories: bird, other vertebrate, invertebrate or plant. We excluded parasites whose life cycle has not been elucidated in vertebrates. Similarly, we recorded information about parasite life cycles, categorising parasites into simple (requiring one host) or complex (requiring multiple hosts). To assess the level of sequence diversity contained in assignments to the same genera, we aligned ASVs assigned to each genus and calculated the proportion of variable sites as well as the pairwise identities amongst them (Figure S2).

### 2.7 Haemosporidian‐Specific Multiplex PCR

Despite the availability of reference sequences in public databases, 18S metabarcoding was unable to detect haemosporidians (*Haemoproteus, Plasmodium* and *Leucocytozoon*). This could be because amplification of mixed template PCRs is biased against AT‐rich sequences (Polz and Cavanaugh 1998) and haemosporidian genomes are known to be AT‐rich (Outlaw and Ricklefs 2010). Therefore, we used a multiplex PCR assay which utilises equimolar concentrations of three sets of primers, targeting different genomic regions of each parasite, these are: PMF, PMR, HMF, HMR, LMF, and LMR (Ciloglu et al. 2019). Each reaction had a final volume of 10 μL and included Qiagen 5X Multiplex PCR Master Mix (Qiagen, Hilden, Germany), 1 μL of each primer at 10 μM (final conc. 0.5 M), 1 μL DNA (ca. 10 ng/μl) and sterile ddH_2_O. The thermal cycling conditions were as follows: 95°C for 15 min; followed by 35 cycles of denaturation at 94°C for 30 s, annealing at 59°C for 90 s, and extension at 72°C for 30 s; with a final extension at 72°C for 10 min. We included one negative (ddH_2_O) and one positive control for every 16 samples. For the positive control, we alternated between using DNA extracts taken from birds with a confirmed *Haemoproteus, Leucocytozoon*, and a mixed *Haemoproteus* and *Plasmodium* infection. Amplified products were resolved using electrophoresis for 1 h at 90 V on a 2% agarose gel containing GelRed gel stain (Biotium Inc., Hayward, CA, United States). The product size was used to infer which genera were present in each sample (*Haemoproteus*: 533 bp, *Plasmodium*: 378 bp, *Leucocytozoon*: 218 bp). For some analyses, we used presence‐absence data for the parasite genera detected per individual by our metabarcoding approach and combined it with the resulting information on haemosporidian incidence.

### 2.8 Statistical Analysis

#### 2.8.1 Alpha and Beta Diversity Analyses

All statistical analyses were conducted in R version 4.4.2 (R Core Team 2024). Throughout, ‘host population’ refers to the three groups compared here (Tasmanian residents, Tasmanian migrants and mainland residents). The two Tasmanian groups belong to the same subspecies but differ in migratory behaviour, whereas mainland residents are a different subspecies (*Z. l. cornwalli*). We therefore use ‘host population’ to indicate both subspecies and migratory status.

To assess whether each host population was adequately sampled to estimate parasite genus richness, we constructed sample size‐based rarefaction curves using individual birds with 1000‐replicate bootstrap confidence intervals (Figure S3). We also estimated incidence‐based asymptotic richness (Chao2) and sample coverage for each host population (Table S3).

To test whether migrant birds carry a greater richness of parasites than their resident conspecifics, we estimated richness for each individual using the standardised ASV data. We estimated observed richness (number of ASVs detected) per sample type (faecal and blood) using the *iNEXT* package (Hsieh et al. 2016). To quantify whether the migrant population had compositionally distinct parasite communities compared to Tasmanian and mainland resident populations, we calculated Local Contribution to Beta Diversity (LCBD). LCBD can be used to quantify proportional contributions to overall beta diversity within the dataset (Legendre and de Cáceres 2013). To compute LCBD, we generated a Jaccard dissimilarity matrix from the standardised ASVs which we converted to presence‐absence data and calculated LCBD using the *Adespatial* package (Dray et al. 2018). Differences in parasite richness and community distinctiveness (LCBD) amongst the populations were assessed using general linear models (GLMs). We included the following factors as possible predictors: sex, date of sampling, host population, and sampling state. To test for differences in parasite sharing between populations according to whether parasites were detected in the blood or faeces, we included an interaction term between host population and sample type.

Observed richness is a count and was subsequently modelled using a negative binomial error distribution; a Poisson error distribution could not be used because the response was overdispersed. LCBD is a bounded proportion, so it was modelled using beta regression with a logit link. We modelled the dispersion parameter on the log scale because the default parameterisation failed to converge. LCBD is calculated relative to the set of samples from which it is computed, this means that values from blood and faecal samples are not on a common scale; the two sample types were therefore modelled separately, and we did not include an interaction term.

Next, we tested for differences in the parasite communities sampled in mainland resident and Tasmanian migrant populations in QLD and NSW. Southern to central‐eastern QLD is considered the limit for silvereye migration (Robertson 1971; Lane 1972; Chan 2005). Therefore, birds sampled in QLD are more likely to have reached their overwintering sites than those sampled in NSW. Consequently, a difference in parasite community richness between the two states (QLD and NSW) could help to elucidate when parasites are gained during the migratory cycle. We calculated observed richness for mainland residents and Tasmanian migrants sampled in each state. We used a GLM with a negative binomial error distribution with observed richness as the response variable. We included the following factors as possible predictors: sex, date of sampling, host population and sampling state. We included an interaction term between host population and sampling state to test whether parasite richness in the Tasmanian migrants differed between the states.

#### 2.8.2 Network‐Based Analysis

We built a bipartite network using the presence‐absence data derived from metabarcoding and the haemosporidian‐specific PCR. The bipartite network enables us to explore whether parasite life cycle complexity influenced patterns of sharing amongst populations. We split the parasite genera into two categories depending on their life cycle complexity. To test whether parasite life cycles had an impact on the richness of parasites infecting the three host populations, we calculated scaled degrees of each bird per parasite life cycle complexity (i.e., the number of links from each bird to parasite genera with simple or complex life cycles, standardised according to the number of total parasite genera in the network). We fitted a GLM using scaled degrees as the response variable, implemented with a binomial error distribution. The following factors were included as possible predictors: parasite life cycle complexity, host population, sex, and date of sampling. We included an interaction term between parasite life cycle complexity and population to test whether parasites with simple life cycles more readily infected migrants.

We assessed the robustness of the interaction between host population and life cycle complexity in three ways. First, we recalculated the model‐averaged estimate of the interaction across model sets defined at differing ΔAICc thresholds (ΔAICc < 2–10). Second, because only three genera were classified as having simple life cycles, we refitted the model omitting each of these genera in turn to test whether the conclusions rely on a single genus. Third, we refitted the model excluding mainland residents, which reduces the analysis to the contrast between Tasmanian migrants and their resident conspecifics.

We took a model selection and averaging approach for all models using the MuMIn package (Barton and Barton 2015). All candidate models were compared based on Akaike's Information Criterion corrected for small sample sizes (AICc), and model selection tables are presented in Tables S4–S8. Models within ΔAICc < 2 of the top‐ranked models were considered equally plausible and we generated model‐averaged predictions across this top model set. We retained this threshold because a difference of less than two AICc units is conventionally taken to indicate models with substantial empirical support (Burnham and Anderson 2002), and because it corresponded to a natural break in the ranking of our candidate models.

Many of our models included two measurements per individual bird. Therefore, we fitted the models as generalised linear mixed models (GLMM) with a random intercept for host individual. We found that the amongst‐individual variance was estimated at zero, and the fixed effects were identical to three decimal places to those of the equivalent GLM (Table S9). This is expected because each individual contributes only two observations, so an individual‐level variance is not distinguishable from binomial sampling variation. We therefore report GLMs with cluster‐robust standard errors based on host individual. This approach accounts for the non‐independence of the repeated measures without requiring the identification of the random intercept (Cameron and Miller 2015). We adopt this approach in all models where two measures are included per individual.

We included sex and date of sampling in all the models. This is because previous work has shown sex‐specific variation in parasite prevalence (Moore and Wilson 2002; Isomursu et al. 2006; Rodriguez et al. 2021; García et al. 2023) and migratory behaviour (Medeiros et al. 2012; Baert et al. 2018; Pedersen et al. 2019). We include date of sampling as a continuous covariate (day of year) to account for potential seasonal variation in parasite dynamics, as we sampled birds from May to July, during the Austral autumn and winter. Parasite prevalence (Deviche et al. 2001; Cosgrove et al. 2008; Tavares‐Dias et al. 2014), load, and diversity have been observed to vary in migrant hosts over time, with late arrivals reported as having both higher (López et al. 2013; Emmenegger et al. 2018) and lower (Mijele et al. 2016) parasite burdens in different systems.

Body condition has also been shown to vary with parasite infection status (Scheuerlein and Ricklefs 2004; Jiménez‐Peñuela et al. 2019) as well as over the course of migration (Jones et al. 2002) and with migratory distance (Møller 2008). Therefore, it is of interest to test whether host body condition impacted individual parasite communities. However, we were unable to include body condition in our GLMs due to missing weight data for 29 individuals. Therefore, to address whether body condition could have impacted our results, we conducted a separate GLM with binomial error distribution using scaled degrees (extracted from the bipartite network, representing proportion of total parasite genera detected per host individual) as the response variable. We included body condition, measured by extracting the residuals from an Ordinary Least Squares regression of body mass (g) by tarsus length (cm) (Brown 1996); sex; population; and date of sampling as possible predictors.

To avoid inclusion of collinear predictor variables in the models, variance inflation factors (VIFs) were computed using the *performance* package to quantify multicollinearity (Lüdecke et al. 2021). VIFs were below five for every predictor in every model, indicating acceptable levels of collinearity for inclusion in the models (Table S10). Interaction terms inflate VIFs; therefore, collinearity was assessed on the additive models. The highest values, for sampling state (4.16) and date of sampling (4.28) in the state‐level model, arise because the two mainland states were sampled sequentially, so the effects of sampling state and date cannot be separated entirely. Model fit was assessed using the performance package (Figures S4–S9).

We tested whether certain parasite genera tended to infect a broader range of host individuals than would be expected by chance. We used the bipartite network to calculate proportional similarity per parasite genera (i.e., specialisation within the network, measured as similarity between host use and availability). We compared the observed proportional similarity of each genus to a null distribution generated for that genus individually. In a fixed‐fixed null model, the number of parasite infections and host individuals remains the same, but the incidence of infection is randomly allocated in the network. We generated 2000 randomisations and, for each genus separately, calculated the mean and standard deviation of its own null distribution. A genus was considered to depart from the null expectation when its observed value fell more than 1.96 standard deviations from its own null mean, corresponding to a 95% interval. Genera detected in fewer than five individuals were excluded because their null distributions are degenerate.

## 3 Results

We identified parasites in 168 individuals comprising 53 Tasmanian residents, 56 Tasmanian‐origin migrants and 59 mainland residents (Figure 1B). Nine of these individuals could not be sexed and were excluded from models including sex as a predictor, leaving 159 individuals (51 Tasmanian residents, 51 Tasmanian‐origin migrants and 57 mainland residents). Eight parasite genera with life cycles characterised in vertebrate hosts were recovered (Table S2): five from metabarcoding analysis and three haemosporidian genera from targeted PCR. Three parasite genera are considered to have simple life cycles (*Eimeria* (López‐Osorio et al. 2020), *Isospora* (Fayer 1980; Box 1981; Parsa et al. 2023), and *Atoxoplasma* (Levine 1982)) and five to have complex life cycles (*Haemoproteus, Plasmodium, Leucocytozoon* (Valkiūnas 2004), *Lankesterella* (Keckeisen et al. 2024), and *Hepatozoon* (Bennett et al. 1992)).

Parasite richness varied amongst the populations (Figure 2). Sample type was retained in every model within the top set, and the interaction between host population and sample type was retained in the best model (Table S11). Population differences in observed richness were therefore largely driven by faecal samples. In blood, neither resident population differed from the migrants. In faeces, Tasmanian residents carried substantially fewer parasite ASVs than migrants (*β* = −1.64, 95% CI −2.29 to −0.98), equivalent to 19% of the migrant richness (95% CI 10%–37%), whereas mainland residents did not differ from migrants (63% of migrant richness, 95% CI 38%–10%). We found no evidence that host population explained variation in LCBD in either sample type: population was retained in one of the five selected models in blood and one of three in faeces, and its averaged estimate was indistinguishable from zero in both (Table S12).

**FIGURE 2:**
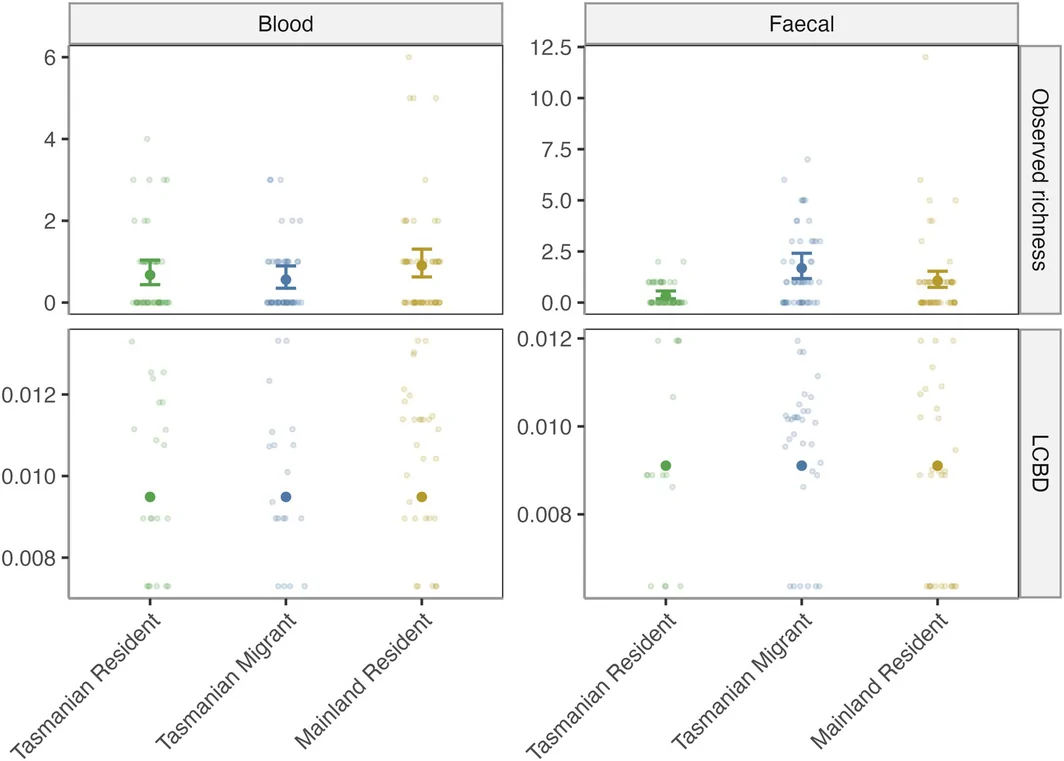
Model‐averaged predictions of observed richness and Local Contribution to Beta Diversity (LCBD) for individual birds from each population (Tasmanian residents, Tasmanian migrants and mainland residents), with 95% cluster‐robust confidence intervals. The raw data are also plotted per individual bird. Richness is shown for blood and faecal samples, which are fitted in a single model while LCBD was fitted in two separate models.

When testing the difference in parasite richness between mainland residents and Tasmanian migrants for each state, we identified four models within AICc < 2 (Table S7). Sampling state was retained in every model within the top set (Table 1). The model‐averaged effect of sampling date indicated a decline in richness over the sampling period (*β* = −0.020, 95% CI −0.034 to −0.005). Richness was higher in the NSW population (Figure 3). However, the top model set did not retain the interaction term between state and population, implying that there was no difference in richness detected in the Tasmanian migrants in each mainland state. Sex was included in some of the top models, although there was some uncertainty about the scale and direction of the effect.

**TABLE 1:** Model‐averaged estimates for a Generalised Linear Model predicting observed richness for Tasmanian migrants (TM) and mainland residents (MR) sampled in New South Wales (NSW) and Queensland (QLD).

Parameter	1	2	3	4	*β*	SE	95% CI
(Intercept)	•	•	•	•	1.496	1.197	(−0.850, 3.841)
Sampling state (QLD)	•	•		•	−0.802	0.274	(−1.338, −0.266)
Sex (male)		•			−0.222	0.242	(−0.696, 0.252)
Sampling date (day of year)			•		−0.020	0.007	(−0.034, −0.005)
Migratory status (migrant)				•	−0.148	0.246	(−0.630, 0.335)
df	3.00	4.00	3.00	4.00			
logLik	−141.80	−141.36	−142.53	−141.61			
AICc	289.92	291.28	291.39	291.78			
Delta	0.00	1.36	1.46	1.85			
Weight	0.42	0.21	0.20	0.17			

*Note:* Coefficient estimates (*β*), standard errors (SE), 95% cluster robust confidence intervals (95% CI) are based on model averaging across the top four models, i.e., the change in small‐sample corrected Akaike Information Criterion was within 2 (ΔAICc < 2). The parameters included in each model are indicated by black dots. The intercept represents the predicted richness when all predictor variables are equal to zero or their reference (baseline) for categorical variables. For each model in the top set, we also present degrees of freedom (df), Log‐likelihood (logLik), AICc, the difference in AICc from the top model (delta) and the Akaike weight, which reflects the relative support for each model.

**FIGURE 3:**
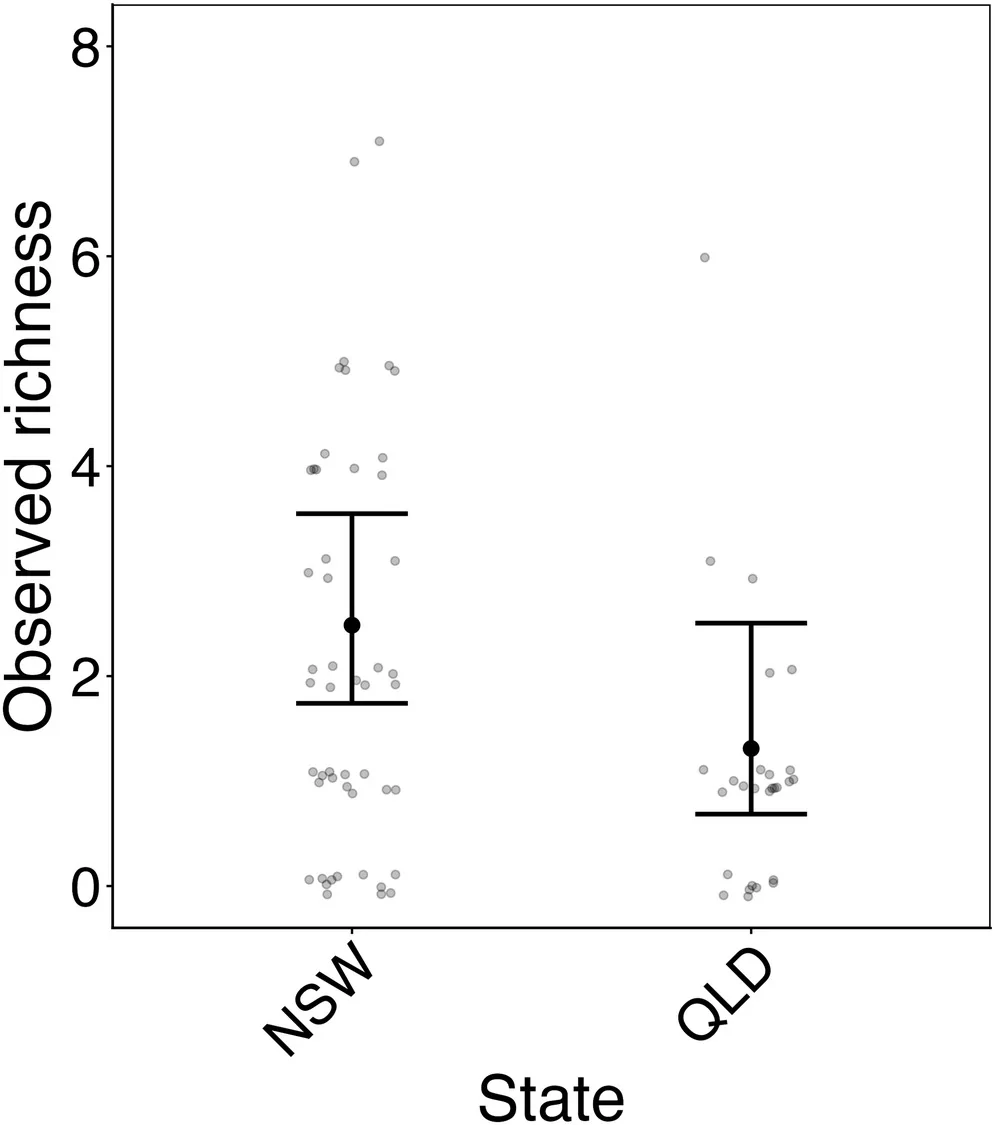
Marginal predicted effects extracted from a generalised linear model with a binomial error distribution testing difference in observed richness for Tasmanian migrants and mainland residents sampled in New South Wales (NSW) and Queensland (QLD). Predictions are averaged across the four models within ΔAICc < 2; error bars are 95% confidence intervals and data for individual birds are plotted as points.

We found four models within < 2 AICc when testing whether parasite life cycle had an impact on richness of parasites infecting the three host populations (Table S8). Life cycle complexity and host population were included in all four of the top models. Simple life cycle parasites infected more individuals than complex life cycle parasites (Figure 4A). The mainland residents carried the most parasite infections, and Tasmanian residents and migrants carried the least (Figure 4B), although there was some uncertainty in estimating this effect (Table 2). The interaction term between host population and life cycle complexity was included in three of the four top models. Its model‐averaged estimate was not sensitive to the threshold used to define the top model set (Table S13). Expressed on the response scale, the predicted proportion of simple life cycle genera infecting an individual was 0.36 (95% CI 0.29–0.45) in Tasmanian migrants compared with 0.23 (0.17–0.31) in Tasmanian residents, a difference of 13.1%, whereas the equivalent values for complex life cycle genera were 0.07 (0.04–0.12) and 0.08 (0.06–0.12), a difference of −1.1% (Figure 4C). The corresponding interaction coefficient was *β* = −0.973 (95% CI −1.792 to −0.155). Excluding mainland residents to compare Tasmanian migrants and their resident conspecific population, the interaction was retained in the single top model and remained clearly supported (*β* = −0.973, 95% CI −1.793 to −0.153; Table S14). Omitting each simple life cycle genus in turn left the direction of the interaction unchanged (*β* between −0.85 and −1.13), although its statistical support depended on *Atoxoplasma*, the most frequently detected of the three (Table S15). Sampling date and sex were each included in one of the top models. We did not find substantial evidence that body condition impacted the parasite infections detected in individual birds (Table S16).

**FIGURE 4:**
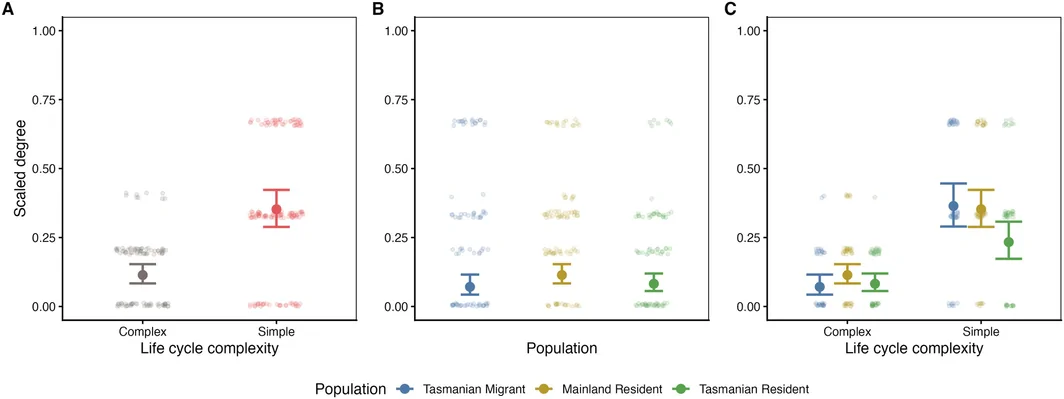
Marginal predicted effects of (A) life cycle complexity, (B) population and (C) the interaction between life cycle complexity and population on scaled degrees (i.e., extracted from the bipartite network, representing proportion of total parasite genera with simple and complex life cycles detected per host individual). Predictions are based on an average of the top four models within < 2 AICc. Error bars represent 95% cluster‐robust confidence intervals and raw data per bird are plotted as points.

**TABLE 2:** Model‐averaged estimates for generalised linear model predicting scaled degrees (extracted from the bipartite network, representing proportion of parasite genera with simple and complex life cycles detected per host individual).

Parameter	1	2	3	4	*β*	SE	95% CI
(Intercept)	•	•	•	•	−2.520	0.342	(−3.190, −1.850)
Life cycle complexity (simple)	•	•	•	•	2.013	0.357	(1.313, 2.712)
Population (Mainland Resident)	•	•	•	•	0.518	0.340	(−0.149, 1.185)
Population (Tasmanian Resident)	•	•	•	•	0.159	0.396	(−0.618, 0.936)
Life cycle complexity (simple) × population (Mainland Resident)	•		•	•	−0.700	0.390	(−1.465, 0.064)
Life cycle complexity (simple) × population (Tasmanian Resident)	•		•	•	−0.973	0.423	(−1.803, −0.144)
Sampling date (day of year)			•		−0.001	0.002	(−0.006, 0.003)
Sex (male)				•	−0.057	0.158	(−0.367, 0.253)
df	6.00	4.00	7.00	7.00			
logLik	−307.37	−310.33	−307.22	−307.31			
AICc	627.01	628.79	628.81	628.97			
Delta	0.00	1.78	1.80	1.96			
Weight	0.46	0.19	0.19	0.17			

*Note:* Coefficient estimates (*β*), standard errors (SE), and 95% cluster robust confidence intervals (95% CI) are based on model averaging across the top four models within ΔAICc < 2. The parameters included in each model are indicated by black dots. The intercept represents the predicted scaled degrees when all predictor variables are equal to zero or their reference (baseline) for categorical variables. For each model in the top set, we also present degrees of freedom (df), Log‐likelihood (logLik), AICc, the difference in AICc from the top model (delta) and the Akaike weight, which reflects the relative support for each model.

The proportional similarity analysis indicated that three parasites (*Lankesterella, Plasmodium*, *and Leucocytozoon*) were less evenly shared across the network than would be expected under a fixed‐fixed null model (Figure 5 and Table S17). These three parasites have complex life cycles.

**FIGURE 5:**
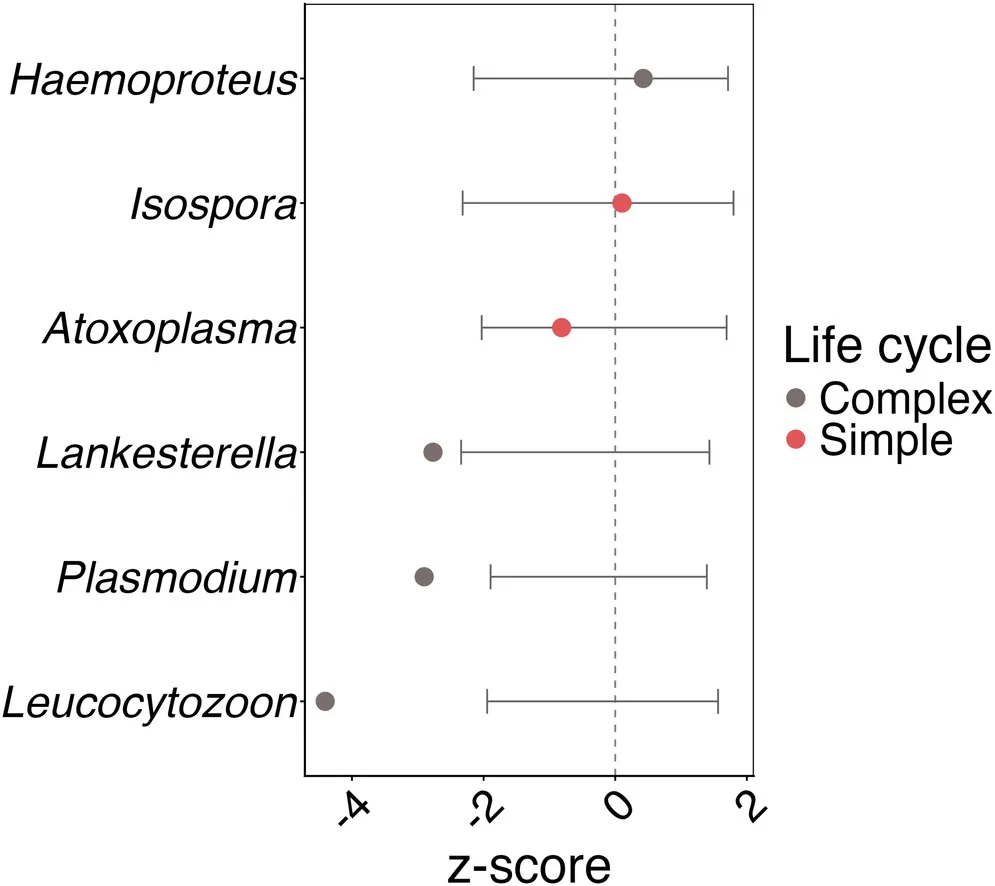
Proportional similarity *z*‐scores for each parasite genera in the bipartite network mapping infections amongst 168 individuals and six parasite genera compared to a fixed‐fixed null model. Error bars indicate the range of *z*‐scores generated in a fixed‐fixed null model per genus and the dashed line is the null expectation. Points show the observed proportional similarity values for each parasite genus and colours indicate parasite life cycle complexity.

## 4 Discussion

By characterising a range of parasites from blood and faecal samples in a partial migrant system, we have revealed that parasite sharing facilitated by migration is influenced by life cycle complexity. While many parasite genera are shared by all three populations, migrant individuals from Tasmania appear to acquire parasite richness during the migratory period, supporting the “migratory exposure hypothesis”. This pattern is largely driven by parasites with simple life cycles which are more readily acquired by Tasmanian migrants than those with complex life cycles. Furthermore, several of the complex life cycle parasites that we detected appear to be less readily shared between the populations. Our results suggest that migrant birds may be gaining parasites from the residents they encounter on the mainland, with hosts sampled in the same mainland states displaying similar levels of parasite richness, despite differing in their migratory behaviour. Collectively, our findings from the partial migrant silvereye system expand our understanding of how transmission dynamics influence parasite sharing across the migratory range.

### 4.1 Parasite Sharing Amongst Migrants and Residents Is Influenced by Parasite Life Cycles

The availability of secondary hosts or vectors may constrain dispersal of parasites with complex life cycles. We detected higher parasite richness in the mainland resident and Tasmanian migrant populations compared to the Tasmanian resident population. Our network analysis confirmed that these richness patterns are driven by the increase in infections of parasites with simple life cycles (particularly *Atoxoplasma* and *Isospora*), which are notably elevated in mainland resident and Tasmanian migrant populations relative to Tasmanian residents. The difference between parasites with simple and complex life cycles was considerably larger than the difference between host populations, indicating that life cycle complexity is the stronger influence on which parasites are shared. This result implies that complex life cycle parasites are less readily shared between hosts during the migratory cycle, a pattern observed previously and often attributed to vector availability (Hellgren et al. 2007; Pulgarín‐R et al. 2019; Soares et al. 2020; Galen et al. 2024).

In the present study, *Leucocytozoon* spp. were less frequently shared amongst silvereye hosts than would be expected by chance. Notably, fewer infections were detected in mainland resident than Tasmanian individuals. Previous work has used *Leucocytozoon* infections to identify immigrant birds for population monitoring (Vysotsky and Valkiūnas 2001). This is possible because *Leucocytozoon* is transmitted by black flies (Diptera: Simuliidae; Pramual et al. 2020; Žiegytė and Bernotienė 2022), which rely on fast flowing water for larval development (Crosskey 1993; Adler et al. 2004) and have a relatively limited dispersal distance of up to 10 km (Walsh et al. 2023). As a result, local‐scale variation in vector availability impacts parasite distributions. The differing habitats of sample collection sites in Tasmania and mainland Australia may thus differentially support *Leucocytozoon* transmission. Assemblages of helminth parasites have similarly been used to track host populations, as their transmission depends on the availability of intermediate hosts such as molluscs or arthropods (Sheehan et al. 2016). These findings highlight that migration is more likely to facilitate the spread of simple life cycle parasites, while distributions of parasites with complex life cycles may be constrained by local ecological conditions that govern the presence and abundance of their intermediate or final hosts (Benesh et al. 2022). Nonetheless, it is evident that several of the complex life cycle parasites detected in our study were broadly shared amongst the three host populations (e.g., *Haemoproteus* and *Hepatozoon*).

Differences in parasite richness became more pronounced when parasites identified in the blood and faecal samples for each host were combined, although differences amongst the three populations were not evident when comparing blood samples alone. The elevated richness detected in faecal samples may reflect differences in parasite life history strategies. Vector‐borne parasites, which are transmitted by bites, spend part of their life cycle in host blood cells, potentially making them more readily detected in blood (Valkiūnas and Atkinson 2020). In contrast, gut‐associated parasites are more likely to rely on environmental transmission, such as excretion and ingestion of transmission stages via faecal‐oral routes (Cevallos and Farthing 1993). Such environmental transmission could better facilitate parasite dispersal during migration (Briscoe et al. 2022; Teitelbaum et al. 2023). Our results indicate that *Atoxoplasma* and *Isospora*, two parasites characterised by faecal‐oral transmission, showed sharing patterns between populations that were indistinguishable from the null expectation, implying that they are widely shared. However, it should be noted that some parasites were detected in both the faeces and blood. This was surprising as their life cycles imply restriction to a specific body compartment. The detection of parasites in host body compartments that are not associated with the parasite life cycle may add to the evidence that gut‐associated DNA from microbes or food can be detected in the blood (Spisak et al. 2013; Damgaard et al. 2015; Zhai et al. 2024). Alternatively, this finding could also reflect limitations in the availability of reference sequences (Hleap et al. 2021; Nichols et al. 2026).

The limitations regarding the availability of reference sequences also restricted the number of parasite taxa that could be classified. Just three of the genera recovered had simple life cycles, and the statistical support for the interaction term depended on *Atoxoplasma*, potentially limiting the extent to which our findings apply to life cycle complexity generally. However, further examination of the 52 ASVs assigned to *Atoxoplasma* and the 22 assigned to *Isospora* revealed that every sequence matched a single reference. The ASVs were also considerably more divergent than would be expected within a genus (Caron et al. 2009). Amongst our ASVs, many pairwise identities were below 95%. The number of simple life cycle genera may therefore reflect the resolution of available reference databases rather than the true taxonomic breadth of these parasite communities, which is likely to be substantially greater. We emphasise that this does not undermine our life cycle complexity categories as the ASV's taxonomic placement within the isosporoid coccidia of passerines was well supported. For isosporoid coccidia such as *Isospora* and *Atoxoplasma*, life cycle is conserved across the clade: they are homoxenous and complete development within a single host (Box 1981; Levine 1982; Barta et al. 2005). These ASVs can thus be attributed confidently to the simple life cycle category, despite their potentially unresolved taxonomic assignments.

Previous efforts to infer the directionality of parasite sharing resulting from migratory host movements have relied on multi‐species comparisons of migratory and resident species at sites where they co‐occur (Pulgarín‐R et al. 2019; de Angeli Dutra, Filion, et al. 2021; Perrin et al. 2025). Such analyses are often confounded by interspecific differences in evolutionary and ecological histories (Binning et al. 2022) and limited by sampling single points in migratory cycles. These complications may underlie contradictory patterns of parasite richness and sharing between migratory and resident hosts (Ricklefs et al. 2017; Teitelbaum et al. 2018; Perrin et al. 2025). In our single host species study, Tasmanian migrants caught on the mainland harboured a substantially richer parasite community as measured by observed richness than Tasmanian resident silvereyes. The ability to compare Tasmanian migrants to their resident ‘counterfactual’ population allows us to infer that Tasmanian migrants gained parasites from mainland residents, as the latter supported richer parasite communities than Tasmanian residents. Taken together, our results provide evidence in support of the “migratory exposure” hypothesis (Altizer et al. 2011) but could be further bolstered by staged sampling along the migratory route of both resident and migrating birds.

The “migratory exposure” hypothesis would further predict that parasite richness should increase with migration distance due to prolonged opportunity for exposure (Gregory 1990; Figuerola and Green 2000). However, we observed a higher overall parasite richness in Tasmanian migrants sampled in NSW compared to QLD, despite the shorter distance travelled by migrants. It is possible that these results are explained by “migratory culling”, i.e., the death of birds that carry heavy parasite loads (Bartel et al. 2011; Slowinski et al. 2018), resulting in the sampled population in QLD having lower parasite richness. However, parasite richness for mainland resident and Tasmanian migrants were comparable in both states. This suggests that the pattern is not due to the loss of heavily infected individuals from the population. Previous work has suggested that host site use, rather than migratory distance, explained changes in helminth parasite richness in Charadriiform birds. We may similarly infer that parasite communities are determined by ecological factors operating at the local scale, causing Tasmanian migrants and mainland residents which are sampled in the same state to be more similar in their parasite richness than individuals belonging to the same population but sampled in different states.

There is a dearth of research exploring the rate at which host individuals gain and lose parasites (Rigaud et al. 2010; Donaldson et al. 2024). It is therefore notable that, despite Tasmanian residents and migrants belonging to the same subspecies and co‐occurring throughout most of the year, parasite communities were more similar between Tasmanian migrants and mainland residents. In addition, Tasmanian residents harboured the fewest parasites of the three populations. This is evidenced by the similarly elevated parasite richness in Tasmanian migrants and mainland residents compared to Tasmanian residents. These results suggest that parasites are gained and lost by migratory birds over short timescales. Parasite life histories may further influence the rate of parasite gain and loss at the host level (Donaldson et al. 2024). For example, complex life cycle parasites could require prolonged periods of incubation, limiting the opportunity for transmission. Future research should consider whether transmission of parasites with complex life cycles is constrained by the length of time that migrants and residents co‐occur.

### 4.2 Conclusions

There are many factors that may influence the likelihood that parasites are shared as a consequence of host migration. Parasite traits can play an important role in facilitating successful sharing. These include parasite specificity (Clark and Clegg 2017; de Angeli Dutra, Moreira Félix, and Poulin 2021), life cycle complexity (as shown here), and life cycle flexibility, such as the ability to use paratenic hosts or to prolong stages of the life cycle (Auld and Tinsley 2015). Virulence could also reduce host survival or migratory success, thereby limiting dispersal (Shaw et al. 2018; Peacock et al. 2020). Furthermore, the time required to clear infections or mount an immune response could reduce the opportunity for parasite sharing amongst hosts (and may also prevent successful detection of the parasite; Buehler et al. 2010; Hegemann et al. 2018). Parasite dispersal may thus be simultaneously influenced by a suite of factors whose varying influences should be considered when interpreting differences in parasite communities across migratory systems.

Our empirical evidence supports the “migratory exposure” hypothesis, whereby migrant birds are exposed to diverse parasite faunas during migration, resulting in a greater parasite richness in migratory birds compared to their resident counterparts. It appears that these patterns are mediated by life cycle complexity, as infections with simple life cycles are particularly common in Tasmanian migrants compared to Tasmanian residents. The tendency for parasites with complex life cycles to be less readily shared throughout the network may reflect the availability of their vectors or secondary hosts. Collectively, our results suggest that migration‐mediated parasite sharing is constrained by the ecology and life history of the parasite. More broadly, our results emphasise the value of partial migrant systems for disentangling the ecological and evolutionary drivers of parasite transmission, and they underscore the importance of integrating host movement, parasite life history, and environmental context in future predictions of parasite spread.

## Author Contributions

S.N., A.E., S.M.C. and B.O. conceptualised and designed the research. S.N., A.E., C.M.Y., J.A., J.T.C., J.L., G.L. and S.M.C. performed the research. This involved organising fieldwork logistics and collecting samples (S.N., A.E., C.M.Y., J.A., J.T.C., J.L., G.L., S.M.C.) as well as conducting lab work (S.N.). S.N. analysed the data and wrote the paper with S.M.C. and B.O. All the authors provided feedback on the manuscript during the drafting process.

## Funding

The study was supported by funding from the Natural Environment Research Council (NERC) through a PhD studentship awarded to S.N. (NE/S007474/1) as well as an American Ornithological Society Student Research Award and a Biology Eurofins Foundation Fund Award (University of Oxford) granted to S.N. Lab work was supported by a student training grant awarded to S.N. and B.O. by the NEOF (NEOF1523). The contribution by A.E. was facilitated by a NERC funded PhD studentship (NE/S007474/1), Heredity Fieldwork Grant (The Genetics Society), Eurofins Foundation Fund, Hesse Research Award (American Ornithological Society), Santander Travel Award (University of Oxford).

## Ethics Statement

Ethical approval was issued to S.M.C. (Griffith University ENV/06/20/AEC) and C.Y. (University of Tasmania 27197). We exported our samples from Australia under permit no. PWS2022‐AU‐001814 and imported samples to the UK under Authorisation No. ITIMP21.1083. We thank the National Parks and Wildlife Service for New South Wales (Scientific Licence SL102647 from Department of Planning, Industry and Environment, NSW, Australia, issued to S.M.C.), Queensland (Research permit WA0042422 from Department of Environment and Science, Queensland, Australia issued to S.M.C.) and Tasmania (Permit No. FA22367 from Department of Natural Resources and Environment, Tasmania, Australia and Scientific Research permit SR‐2022‐5283, Hobart City Council, Tasmania, Australia issued to C.Y.) for sampling permissions. We recognise ABBBS for issuing a project licence to S.M.C.

## Conflicts of Interest

The authors declare no conflicts of interest.

## Supporting information


**Figure S1:** Illustration of faecal sampling protocol: flat‐bottomed paper bag lined with sterile weigh‐boats covered with wire mesh, ventilated by puncturing the sides of the paper bag. Pegs were used to secure the top of the bag.
**Figure S2:** Within‐genus sequence divergence amongst amplicon sequence variants (ASVs) assigned to *Atoxoplasma*, *Isospora* and *Eimeria*, the three simple life cycle genera detected by metabarcoding. ASVs assigned to each genus were aligned to a single reference sequence; the histogram shows the distribution of pairwise percentage identity amongst ASVs within each genus. The dashed line marks 97% identity, a conventional species‐level threshold.
**Figure S3:** Sample‐size‐based rarefaction (solid lines) and extrapolation (dashed lines) curves of parasite genus richness for each host population. Shaded bands show 95% confidence intervals from 1000 bootstrap replicates.
**Figure S4:** Model fit diagnostic panel produced using the performance package (Lüdecke et al. 2021) for the generalised linear model with a negative binomial error distribution predicting observed richness of parasite ASVs, according to host population (POP), sample type (blood, faecal; TYPE), sex and date of sampling (DOY), including a population by sample type interaction term (POP:TYPE).
**Figure S5:** Model fit diagnostic panel produced using the performance package (Lüdecke et al. 2021) for the beta regression predicting local contribution to beta diversity (LCBD) from host population, sex and date of sampling, in blood samples.
**Figure S6:** Model fit diagnostic panel produced using the performance package (Lüdecke et al. 2021) for the beta regression predicting local contribution to beta diversity (LCBD) from host population, sex and date of sampling, in faecal samples.
**Figure S7:** Model fit diagnostic panel produced using the performance package (Lüdecke et al. 2021) for the generalised linear model with a negative binomial error distribution predicting observed parasite genus richness according to sampling state (STATE), migratory status (STATUS), sex and date of sampling (DOY) in Tasmanian migrants and mainland residents, including a state by status interaction term (STATE:STATUS).
**Figure S8:** Model fit diagnostic panel produced using the performance package (Lüdecke et al. 2021) for the generalised linear model predicting scaled degrees extracted from a bipartite network (i.e., the number of links to each parasite genus with simple and complex life cycles per host individual, standardised for total parasite genera), including life cycle complexity (LCC), host population (POP), sex, day of year (DOY), and an interaction between host population and life cycle complexity (POP:LCC).
**Figure S9:** Model fit diagnostic panel produced using the performance package (Lüdecke et al. 2021) for generalised linear model with a binomial error distribution predicting scaled degrees (extracted from a bipartite network, representing parasite infections standardised according to total parasites detected) according to body condition, population, sex and date of sampling.
**Table S1:** List of accession numbers used to build the maximum‐likelihood phylogenetic tree inferred using RAxML‐NG. These accession numbers were adapted from Rapp and Wolf (2024).
**Table S2:** Parasites identified in 168 silvereye individuals comprising 53 Tasmanian residents, 56 Tasmanian migrants and 59 mainland residents with information about their previously recorded hosts and life cycle complexity.
**Table S3:** Sampling completeness of parasite genus richness per host population. Chao2 estimates are incidence‐based, with 95% confidence intervals from 1000 bootstrap replicates. Sample coverage is the Chao estimator for incidence data.
**Table S4:** Model selection results for a generalised linear model with a negative binomial error distribution predicting observed richness of parasite amplicon sequence variants for mainland residents and Tasmanian migrants sampled in New South Wales (NSW) and Queensland (QLD), treating blood and faecal samples from each bird as a single community. Each row represents an individual model and includes parameter estimates (intercept and coefficients), degrees of freedom (df), log‐likelihood (logLik), Akaike Information Criterion corrected for small sample size (AICc), delta AICc (ΔAICc), and Akaike weights. NA values indicate that the parameter was not included in the model.
**Table S5:** Model selection results for a beta regression predicting Local Contribution to Beta Diversity (LCBD) in blood samples. Each row represents an individual model and includes parameter estimates (intercept and coefficients), degrees of freedom (df), log‐likelihood (logLik), Akaike Information Criterion corrected for small sample size (AICc), delta AICc (ΔAICc), and Akaike weights. NA values indicate that the parameter was not included in the model.
**Table S6:** Model selection results for a beta regression predicting Local Contribution to Beta Diversity (LCBD) in faecal samples. Each row represents an individual model and includes parameter estimates (intercept and coefficients), degrees of freedom (df), log‐likelihood (logLik), Akaike Information Criterion corrected for small sample size (AICc), delta AICc (ΔAICc), and Akaike weights. NA values indicate that the parameter was not included in the model.
**Table S7:** Model selection results for a generalised linear model with a negative binomial error distribution predicting observed richness for Tasmanian migrants and mainland residents sampled in New South Wales (NSW) and Queensland (QLD). Each row represents an individual model and includes parameter estimates (intercept and coefficients), degrees of freedom (df), log‐likelihood (logLik), Akaike Information Criterion corrected for small sample size (AICc), delta AICc (ΔAICc), and Akaike weights. NA values indicate that the parameter was not included in the model.
**Table S8:** Model selection results for GLM predicting scaled degrees from simple and complex life cycle parasites in a bipartite network. Each row represents an individual model and includes parameter estimates (intercept and coefficients), degrees of freedom (df), log‐likelihood (logLik), Akaike Information Criterion corrected for small sample size (AICc), delta AICc (ΔAICc), and Akaike weights. NA values indicate that the parameter was not included in the model.
**Table S9:** Comparison of the generalised linear model (GLM) reported in the main text with the equivalent generalised linear mixed model (GLMM) including a random intercept for host individual. The variance amongst individuals was estimated as 0 (SD < 0.0001), so the two models are equivalent and the coefficients are identical to three decimal places. Standard errors are model‐based in both cases.
**Table S10:** Pairwise Variance inflation factors (VIFs) calculated for each predictor variable considered for inclusion in the generalised linear models.
**Table S11:** Model‐averaged estimates for a generalised linear model with a negative binomial error distribution predicting observed richness of parasite amplicon sequence variants for mainland residents and Tasmanian migrants sampled in New South Wales (NSW) and Queensland (QLD), treating blood and faecal samples from each bird as a single community. Coefficient estimates (*β*), standard errors (SE) and 95% cluster‐robust confidence intervals (95% CI) are model‐averaged across the two models within ΔAICc < 2; standard errors are clustered on host individual. The parameters included in each model are indicated by black dots. Full model selection details are given in Table S4.
**Table S12:** Beta regression model‐averaged estimates for Local Contribution to Beta Diversity (LCBD) in blood and faecal samples, modelled separately because LCBD is calculated relative to its own sample set. Coefficient estimates (*β*), standard errors (SE) and 95% confidence intervals (95% CI) are averaged across all models within ΔAICc < 2 of the best model for each sample type (five models for blood, three for faecal). ‘(phi)’ rows give the beta regression's dispersion parameter, modelled on the log scale and estimated in every model. The parameters included in each model are indicated by black dots. Full model selection details are given in Tables S5 and S6.
**Table S13:** Support for the host population × life cycle complexity interaction across model sets defined by different ΔAICc cut‐offs. The summed Akaike weight is the total weight of models within the set that contain the interaction, renormalised across that set.
**Table S14:** Model of scaled degrees fitted with mainland residents excluded. Estimates are from the top model according to ΔAIC < 2, with cluster‐robust standard errors clustered on host individual.
**Table S15:** Leave‐one‐genus‐out refits. The model was refitted omitting each simple life cycle genus in turn. The coefficient shown is the life cycle complexity (Simple) × host population (Tasmanian Resident) interaction, model‐averaged across models within ΔAICc < 2 with cluster‐robust standard errors.
**Table S16:** Model‐averaged estimates for GLM predicting scaled degrees (proportion of degrees linking parasite genera to host individuals extracted from bipartite network). Coefficient estimates (*β*), standard errors (SE) and 95% cluster robust confidence intervals (95% CI) are based on model averaging across the top two models (ΔAICc < 2). The parameters included in each model are indicated by black dots. The intercept represents the predicted scaled degrees when all predictor variables are equal to zero or their reference (baseline) for categorical variables. For each model in the top set, we also present degrees of freedom (df), Log‐likelihood (logLik), small‐sample corrected Akaike Information Criterion (AICc), the difference in AICc from the top model (delta) and the Akaike weight, which reflects the relative support for each model.
**Table S17:** Proportional similarity of each parasite genus compared to its own fixed‐fixed null distribution, generated from 2000 randomisations of the observed network. A genus was considered to depart from the null expectation where its observed value fell more than 1.96 standard deviations from the mean of its own null distribution. Genera detected in fewer than five individuals were excluded, because their null distributions are degenerate.

## Data Availability

The raw sequence data that support the findings of this study are openly available in the NCBI Sequence Read Archive (SRA; https://www.ncbi.nlm.nih.gov/sra). Reads from 67 blood samples are deposited under BioProject PRJNA1415190, and reads from the remaining 101 blood samples and all 165 faecal samples are deposited under BioProject PRJNA1554368.
