# Supplementary material for: Life Cycle Complexity Shapes Parasite Sharing Amongst Migratory and Resident Hosts
Source: Mol Ecol. 2026 Nov 10;35(21):e70657. doi: 10.1111/mec.70657 (PMC13752385; doi:10.1111/mec.70657)
Supplement: Supplementary file 1 [file MEC-35-e70657-s001.docx]

**Figure S1.** Illustration of faecal sampling protocol: flat-bottomed paper bag lined with sterile weigh-boats covered with wire mesh, ventilated by puncturing the sides of the paper bag. Pegs were used to secure the top of the bag.

**Figure S2**. Within-genus sequence divergence among amplicon sequence variants (ASVs) assigned to *Atoxoplasma*, *Isospora* and *Eimeria*, the three simple life cycle genera detected by metabarcoding. ASVs assigned to each genus were aligned to a single reference sequence; the histogram shows the distribution of pairwise percentage identity among ASVs within each genus. The dashed line marks 97% identity, a conventional species-level threshold.

**Figure S3**. Sample-size-based rarefaction (solid lines) and extrapolation (dashed lines) curves of parasite genus richness for each host population. Shaded bands show 95% confidence intervals from 1000 bootstrap replicates.

**Figure S4.** Model fit diagnostic panel produced using the performance package (Lüdecke et al., 2021) for the generalised linear model with a negative binomial error distribution predicting observed richness of parasite ASVs, according to host population (POP), sample type (blood, faecal; TYPE), sex and date of sampling (DOY), including a population by sample type interaction term (POP:TYPE).

**Figure S5.** Model fit diagnostic panel produced using the performance package (Lüdecke et al., 2021) for the beta regression predicting local contribution to beta diversity (LCBD) from host population, sex and date of sampling, in blood samples.

**Figure S6.** Model fit diagnostic panel produced using the performance package (Lüdecke et al., 2021) for the beta regression predicting local contribution to beta diversity (LCBD) from host population, sex and date of sampling, in faecal samples.

**Figure S7**. Model fit diagnostic panel produced using the performance package (Lüdecke et al., 2021) for the generalised linear model with a negative binomial error distribution predicting observed parasite genus richness according to sampling state (STATE), migratory status (STATUS), sex and date of sampling (DOY) in Tasmanian migrants and mainland residents, including a state by status interaction term (STATE:STATUS).

**Figure S8**. Model fit diagnostic panel produced using the performance package (Lüdecke et al., 2021) for the generalised linear model predicting scaled degrees extracted from a bipartite network (i.e. the number of links to each parasite genus with simple and complex life cycles per host individual, standardised for total parasite genera), including life cycle complexity (LCC), host population (POP), sex, day of year (DOY), and an interaction between host population and life cycle complexity (POP:LCC).

**Figure S9**. Model fit diagnostic panel produced using the performance package (Lüdecke *et al.,* 2021) for generalised linear model with a binomial error distribution predicting scaled degrees (extracted from a bipartite network, representing parasite infections standardised according to total parasites detected) according to body condition, population, sex and date of sampling.

**Table S1.** List of accession numbers used to build the maximum-likelihood phylogenetic tree inferred using RAxML-NG. These accession numbers were adapted from Rapp and Wolf (2024).

| Accession | Genera |
| --- | --- |
| AF029763.1 | *Balantidium* |
| AF043355.1 | *Bangia* |
| AF043364.2 | *Pseudobangia* |
| L26189.1 | *Erythrotrichia* |
| AF139462.1 | *Rhodochaete* |
| U25637.1 | *Ichthyophonus* |
| L29455.1 | *Choanoflagellate* |
| AF394528.1 | *Microsporidium* |
| U33180.1 | *Psorospermium* |
| D63696.1 | *Aspergillus* |
| X04971.1 | *Neurospora* |
| X54864.1 | *Pododpora* |
| U07367.1 | *Plasmodium* |
| X12708.1 | *Pneumocystis* |
| X15590.1 | *Fragaria* |
| GU476424.1 | *Eucalyptus* |
| X74753.1 | *Genicularina* |
| KM020091.1 | *Uronema* |
| X77692.1 | *Euglypha* |
| U09048.1 | *Alexandrium* |
| X59604.1 | *Babesia* |
| EF551335.1 | *Babesia* |
| KT361081.1 | *Cytauxzoon* |
| MT904044.1 | *Cytauxzoon* |
| MT904037.1 | *Cytauxzoon* |
| XR_009718807.1 | *Theileria* |
| XR_696411.1 | *Theileria* |
| FJ717705.1 | *Babesia* |
| DQ200887.1 | *Babesia* |
| MH615006.1 | *Hepatozoon* |
| MG136687.1 | *Hepatozoon* |
| KF939628.1 | *Hepatozoon* |
| KX507249.1 | *Haemogregarina* |
| KX507246.1 | *Haemogregarina* |
| U16159.1 | *Neospora* |
| EF472967.1 | *Toxoplasma* |
| XR_001974082.1 | *Toxoplasma* |
| L37415.1 | *Toxoplasma* |
| XR_003828656.1 | *Besnoitia* |
| AY665399.2 | *Besnoitia* |
| GU479632.1 | *Besnoitia* |
| KU341121.1 | *Sarcocystis* |
| MH590230.1 | *Sarcocystis* |
| KU341123.1 | *Sarcocystis* |
| KU180248.1 | *Lankesterella* |
| MF167545.1 | *Lankesterella* |
| AY331569.1 | *Atoxoplasma* |
| KT224380.1 | *Isospora* |
| KY801685.1 | *Isospora* |
| AF080614.1 | *Eimeria* |
| DQ136185.1 | *Eimeria* |
| EF210323.1 | *Eimeria* |
| AF080611.1 | *Lankesterella* |
| L31922.1 | *Babesia* |
| AF129883.1 | *Ophriocystis* |
| AJ535150.1 | *Achnanthes* |
| AJ535158.1 | *Amphora* |
| AF374482.2 | *Thalassiosira* |
| AJ535152.1 | *Pseudogomphonema* |
| AJ535155.1 | *Sellaphora* |
| X77703.1 | *Rhaphoneis* |
| AJ535140.1 | *Thalassionema* |
| X74752.1 | *Staurastrum* |
| AY216906.1 | *Grammatophora* |
| Y10569.1 | *Lithodesmium* |
| X85396.1 | *Thalassiosira* |
| X85395.1 | *Skeletonema* |
| AF374481.2 | *Thalassiosira* |
| AF525667.1 | *Lampriscus* |
| AJ535188.1 | *Pleurosira* |
| AF123595.1 | *Bolidomonas* |
| X85404.2 | *Aulacoseira* |
| AJ535186.1 | *Aulacoseira* |
| AJ535175.1 | *Leptocylindrus* |
| L27634.1 | *Labyrinthuloides* |
| X85399.2 | *Lauderia* |
| M54939.1 | *Lagenidium* |
| X85402.2 | *Melosira* |
| AF164133.1 | *Gastrostyla* |
| AF164128.1 | *Pleurotricha* |
| AF164127.1 | *Paraurostyla* |
| AF164135.1 | *Cyrtohymena* |
| X53486.1 | *Oxytricha* |
| AF164122.1 | *Oxytricha* |
| U23936.1 | *Allomyces* |
| Z14142.1 | *Palmaria* |
| AF132294.1 | *Balbiania* |
| AF026040.1 | *Audouinella* |
| AF342743.1 | *Nemalionopsis* |
| L26190.1 | *Gelidium* |
| U33133.1 | *Halymenia* |
| U09621.1 | *Rhodymenia* |
| U09618.1 | *Plocamiocolax* |
| M33639.1 | *Gracilariopsis* |
| L26183.1 | *Ceramium* |
| Z14139.1 | *Ahnfeltia* |
| AF006089.1 | *Rhodogorgon* |
| Z36893.1 | *Crossodonthina* |
| K03432.1 | *Homo* |
| NR_046237.3 | *Rattus* |
| XR_010350767.1 | *Passer* |
| L23799.1 | *Nucleospora* |
| KJ736741.1 | *Gregarina* |
| LR814087.1 | *Gregarina* |
| AY117418.1 | *Jakoba* |
| AY117417.1 | *Reclinomonas* |
| KU664396.1 | *Gregarina* |
| M19172.1 | *Plasmodium* |
| MK650492.1 | *Plasmodium* |
| MK650552.1 | *Plasmodium* |
| U07367.1 | *Plasmodium* |
| MK650858.1 | *Leucocytozoon* |

**Table S2.** Parasites identified in 168 silvereye individuals comprising 53 Tasmanian residents, 56 Tasmanian migrants and 59 mainland residents with information about their previously recorded hosts and life cycle complexity.

| Genera | Host | Life cycle | Source |
| --- | --- | --- | --- |
| *Eimeria* | Vertebrates | Simple | López-Osorio *et al.* (2020) |
| *Isospora* | Vertebrates | Simple | Fayer (1980); Box (1981); Parsa *et al.* (2023) |
| *Atoxoplasma* | Vertebrates | Simple | Levine (1982) |
| *Haemoproteus* | Vertebrates | Complex | Valkiunas (2004) |
| *Plasmodium* | Vertebrates | Complex | Valkiunas (2004) |
| *Leucocytozoon* | Vertebrates | Complex | Valkiunas (2004) |
| *Lankesterella* | Vertebrates | Complex | Keckeisen *et al.* (2024) |
| *Hepatozoon* | Vertebrates | Complex | Bennett *et al.* (1992) |

**Table S3.** Sampling completeness of parasite genus richness per host population. Chao2 estimates are incidence-based, with 95% confidence intervals from 1000 bootstrap replicates. Sample coverage is the Chao estimator for incidence data.

| **Population** | **S observed** | **Chao2** | **95% CI** | **Coverage** |
| --- | --- | --- | --- | --- |
| Mainland Resident | 7 | 7.00 | (6.00, 7.98) | 99.0% |
| Tasmanian Migrant | 7 | 8.96 | (5.00, 9.95) | 97.5% |
| Tasmanian Resident | 5 | 5.00 | (4.00, 5.00) | 100% |

**Table S4.** Model selection results for a generalised linear model with a negative binomial error distribution predicting observed richness of parasite amplicon sequence variants for mainland residents and Tasmanian migrants sampled in New South Wales (NSW) and Queensland (QLD), treating blood and faecal samples from each bird as a single community. Each row represents an individual model and includes parameter estimates (intercept and coefficients), degrees of freedom (df), log-likelihood (logLik), Akaike Information Criterion corrected for small sample size (AICc), delta AICc (ΔAICc), and Akaike weights. NA values indicate that the parameter was not included in the model.

|  | (Intercept) | Sampling Date | Population | Sample type | Sex | Population × Sample type | df | logLik | AICc | delta | weight |
| --- | --- | --- | --- | --- | --- | --- | --- | --- | --- | --- | --- |
| 23 | -0.60 | NA | + | + | NA | + | 7 | -401.37 | 817.09 | 0.00 | 0.46 |
| 24 | -0.12 | 0.00 | + | + | NA | + | 8 | -400.84 | 818.13 | 1.04 | 0.27 |
| 31 | -0.58 | NA | + | + | + | + | 8 | -401.35 | 819.15 | 2.06 | 0.16 |
| 32 | -0.10 | 0.00 | + | + | + | + | 9 | -400.83 | 820.23 | 3.14 | 0.10 |
| 7 | -0.08 | NA | + | + | NA | NA | 5 | -409.27 | 828.73 | 11.64 | 0.00 |
| 3 | 0.09 | NA | + | NA | NA | NA | 4 | -410.44 | 829.01 | 11.92 | 0.00 |
| 8 | 0.38 | 0.00 | + | + | NA | NA | 6 | -408.82 | 829.90 | 12.81 | 0.00 |
| 4 | 0.58 | 0.00 | + | NA | NA | NA | 5 | -409.93 | 830.06 | 12.97 | 0.00 |
| 15 | -0.08 | NA | + | + | + | NA | 6 | -409.27 | 830.80 | 13.71 | 0.00 |
| 11 | 0.10 | NA | + | NA | + | NA | 5 | -410.44 | 831.08 | 13.99 | 0.00 |
| 16 | 0.38 | 0.00 | + | + | + | NA | 7 | -408.81 | 831.98 | 14.89 | 0.00 |
| 12 | 0.58 | 0.00 | + | NA | + | NA | 6 | -409.93 | 832.13 | 15.04 | 0.00 |
| 5 | -0.33 | NA | NA | + | NA | NA | 3 | -415.20 | 836.47 | 19.39 | 0.00 |
| 1 | -0.15 | NA | NA | NA | NA | NA | 2 | -417.08 | 838.19 | 21.10 | 0.00 |
| 6 | -0.15 | 0.00 | NA | + | NA | NA | 4 | -415.12 | 838.36 | 21.27 | 0.00 |
| 13 | -0.33 | NA | NA | + | + | NA | 4 | -415.20 | 838.53 | 21.44 | 0.00 |
| 2 | 0.03 | 0.00 | NA | NA | NA | NA | 3 | -417.00 | 840.07 | 22.98 | 0.00 |
| 9 | -0.14 | NA | NA | NA | + | NA | 3 | -417.08 | 840.23 | 23.14 | 0.00 |
| 14 | -0.16 | 0.00 | NA | + | + | NA | 5 | -415.12 | 840.42 | 23.33 | 0.00 |
| 10 | 0.03 | 0.00 | NA | NA | + | NA | 4 | -417.00 | 842.12 | 25.03 | 0.00 |

**Table S5.** Model selection results for a beta regression predicting Local Contribution to Beta Diversity (LCBD) in blood samples. Each row represents an individual model and includes parameter estimates (intercept and coefficients), degrees of freedom (df), log-likelihood (logLik), Akaike Information Criterion corrected for small sample size (AICc), delta AICc (ΔAICc), and Akaike weights. NA values indicate that the parameter was not included in the model.

|  | (Intercept) | Sampling Date | Population | Sex | df | logLik | AICc | delta | weight |
| --- | --- | --- | --- | --- | --- | --- | --- | --- | --- |
| 6 | -4.87 | 0.00 | NA | + | 4 | 340.37 | -672.12 | 0.00 | 0.26 |
| 2 | -4.83 | 0.00 | NA | NA | 3 | 339.18 | -672.00 | 0.12 | 0.24 |
| 5 | -4.64 | NA | NA | + | 3 | 338.65 | -670.94 | 1.18 | 0.14 |
| 1 | -4.59 | NA | NA | NA | 2 | 337.37 | -670.56 | 1.56 | 0.12 |
| 4 | -4.93 | 0.00 | + | NA | 5 | 340.58 | -670.23 | 1.90 | 0.10 |
| 8 | -4.96 | 0.00 | + | + | 6 | 341.56 | -669.79 | 2.34 | 0.08 |
| 3 | -4.61 | NA | + | NA | 4 | 338.09 | -667.56 | 4.56 | 0.03 |
| 7 | -4.65 | NA | + | + | 5 | 339.19 | -667.45 | 4.68 | 0.03 |

**Table S6.** Model selection results for a beta regression predicting Local Contribution to Beta Diversity (LCBD) in faecal samples. Each row represents an individual model and includes parameter estimates (intercept and coefficients), degrees of freedom (df), log-likelihood (logLik), Akaike Information Criterion corrected for small sample size (AICc), delta AICc (ΔAICc), and Akaike weights. NA values indicate that the parameter was not included in the model.

|  | (Intercept) | Sampling Date | Population | Sex | df | logLik | AICc | delta | weight |
| --- | --- | --- | --- | --- | --- | --- | --- | --- | --- |
| 1 | -4.69 | NA | NA | NA | 2 | 365.80 | -727.43 | 0.00 | 0.36 |
| 5 | -4.71 | NA | NA | + | 3 | 366.10 | -725.87 | 1.56 | 0.17 |
| 3 | -4.65 | NA | + | NA | 4 | 367.16 | -725.75 | 1.68 | 0.16 |
| 2 | -4.66 | 0.00 | NA | NA | 3 | 365.82 | -725.31 | 2.12 | 0.13 |
| 7 | -4.67 | NA | + | + | 5 | 367.29 | -723.72 | 3.71 | 0.06 |
| 6 | -4.68 | 0.00 | NA | + | 4 | 366.13 | -723.71 | 3.72 | 0.06 |
| 4 | -4.59 | 0.00 | + | NA | 5 | 367.24 | -723.63 | 3.80 | 0.05 |
| 8 | -4.61 | 0.00 | + | + | 6 | 367.39 | -721.56 | 5.87 | 0.02 |

**Table S7.** Model selection results for a generalised linear model with a negative binomial error distribution predicting observed richness for Tasmanian migrants and mainland residents sampled in New South Wales (NSW) and Queensland (QLD). Each row represents an individual model and includes parameter estimates (intercept and coefficients), degrees of freedom (df), log-likelihood (logLik), Akaike Information Criterion corrected for small sample size (AICc), delta AICc (ΔAICc), and Akaike weights. NA values indicate that the parameter was not included in the model.

|  | (Intercept) | Sampling Date | Sex | State | Migratory status | State × Migratory status | df | logLik | AICc | delta | weight |
| --- | --- | --- | --- | --- | --- | --- | --- | --- | --- | --- | --- |
| 5 | 0.91 | NA | NA | + | NA | NA | 3 | -141.80 | 289.92 | 0.00 | 0.23 |
| 7 | 1.03 | NA | + | + | NA | NA | 4 | -141.36 | 291.28 | 1.36 | 0.12 |
| 2 | 3.64 | -0.02 | NA | NA | NA | NA | 3 | -142.53 | 291.39 | 1.46 | 0.11 |
| 13 | 0.97 | NA | NA | + | + | NA | 4 | -141.61 | 291.78 | 1.85 | 0.09 |
| 6 | 1.55 | 0.00 | NA | + | NA | NA | 4 | -141.74 | 292.04 | 2.12 | 0.08 |
| 29 | 0.90 | NA | NA | + | + | + | 5 | -140.96 | 292.76 | 2.84 | 0.06 |
| 15 | 1.11 | NA | + | + | + | NA | 5 | -141.12 | 293.09 | 3.17 | 0.05 |
| 4 | 3.62 | -0.02 | + | NA | NA | NA | 4 | -142.37 | 293.29 | 3.37 | 0.04 |
| 10 | 3.64 | -0.02 | NA | NA | + | NA | 4 | -142.48 | 293.52 | 3.59 | 0.04 |
| 8 | 1.20 | 0.00 | + | + | NA | NA | 5 | -141.36 | 293.56 | 3.64 | 0.04 |
| 14 | 1.37 | 0.00 | NA | + | + | NA | 5 | -141.59 | 294.02 | 4.10 | 0.03 |
| 31 | 1.04 | NA | + | + | + | + | 6 | -140.50 | 294.19 | 4.27 | 0.03 |
| 30 | 2.54 | -0.01 | NA | + | + | + | 6 | -140.67 | 294.55 | 4.62 | 0.02 |
| 12 | 3.62 | -0.02 | + | NA | + | NA | 5 | -142.30 | 295.44 | 5.52 | 0.01 |
| 16 | 0.93 | 0.00 | + | + | + | NA | 6 | -141.12 | 295.44 | 5.52 | 0.01 |
| 1 | 0.69 | NA | NA | NA | NA | NA | 2 | -146.12 | 296.39 | 6.47 | 0.01 |
| 32 | 2.09 | -0.01 | + | + | + | + | 7 | -140.39 | 296.39 | 6.47 | 0.01 |
| 3 | 0.83 | NA | + | NA | NA | NA | 3 | -145.66 | 297.64 | 7.72 | 0.00 |
| 9 | 0.75 | NA | NA | NA | + | NA | 3 | -145.97 | 298.27 | 8.35 | 0.00 |
| 11 | 0.90 | NA | + | NA | + | NA | 4 | -145.47 | 299.49 | 9.57 | 0.00 |

**Table S8.** Model selection results for GLM predicting scaled degrees from simple and complex life cycle parasites in a bipartite network. Each row represents an individual model and includes parameter estimates (intercept and coefficients), degrees of freedom (df), log-likelihood (logLik), Akaike Information Criterion corrected for small sample size (AICc), delta AICc (ΔAICc), and Akaike weights. NA values indicate that the parameter was not included in the model.

|  | (Intercept) | Sampling Date | Life cycle complexity | Population | Sex | Life cycle complexity × Population | df | logLik | AICc | delta | weight |
| --- | --- | --- | --- | --- | --- | --- | --- | --- | --- | --- | --- |
| 23 | -2.64 | NA | + | + | NA | + | 6 | -307.37 | 627.01 | 0.00 | 0.34 |
| 7 | -2.27 | NA | + | + | NA | NA | 4 | -310.33 | 628.79 | 1.78 | 0.14 |
| 24 | -2.40 | 0.00 | + | + | NA | + | 7 | -307.22 | 628.81 | 1.80 | 0.14 |
| 31 | -2.60 | NA | + | + | + | + | 7 | -307.31 | 628.97 | 1.96 | 0.13 |
| 8 | -2.04 | 0.00 | + | + | NA | NA | 5 | -310.19 | 630.57 | 3.56 | 0.06 |
| 15 | -2.24 | NA | + | + | + | NA | 5 | -310.27 | 630.73 | 3.72 | 0.05 |
| 32 | -2.37 | 0.00 | + | + | + | + | 8 | -307.16 | 630.79 | 3.78 | 0.05 |
| 3 | -2.31 | NA | + | NA | NA | NA | 2 | -313.81 | 631.65 | 4.64 | 0.03 |
| 16 | -2.01 | 0.00 | + | + | + | NA | 6 | -310.13 | 632.53 | 5.52 | 0.02 |
| 11 | -2.27 | NA | + | NA | + | NA | 3 | -313.71 | 633.49 | 6.48 | 0.01 |
| 4 | -2.20 | 0.00 | + | NA | NA | NA | 3 | -313.77 | 633.62 | 6.60 | 0.01 |
| 12 | -2.16 | 0.00 | + | NA | + | NA | 4 | -313.67 | 635.48 | 8.47 | 0.00 |
| 5 | -1.51 | NA | NA | + | NA | NA | 3 | -362.56 | 731.19 | 104.18 | 0.00 |
| 6 | -1.29 | 0.00 | NA | + | NA | NA | 4 | -362.43 | 732.98 | 105.97 | 0.00 |
| 13 | -1.47 | NA | NA | + | + | NA | 4 | -362.50 | 733.12 | 106.11 | 0.00 |
| 1 | -1.54 | NA | NA | NA | NA | NA | 1 | -365.74 | 733.50 | 106.49 | 0.00 |
| 14 | -1.27 | 0.00 | NA | + | + | NA | 5 | -362.37 | 734.93 | 107.92 | 0.00 |
| 9 | -1.51 | NA | NA | NA | + | NA | 2 | -365.65 | 735.34 | 108.33 | 0.00 |
| 2 | -1.44 | 0.00 | NA | NA | NA | NA | 2 | -365.71 | 735.46 | 108.44 | 0.00 |
| 10 | -1.41 | 0.00 | NA | NA | + | NA | 3 | -365.62 | 737.32 | 110.31 | 0.00 |

**Table S9.** Comparison of the generalised linear model (GLM) reported in the main text with the equivalent generalised linear mixed model (GLMM) including a random intercept for host individual. The variance among individuals was estimated as 0 (SD < 0.0001), so the two models are equivalent and the coefficients are identical to three decimal places. Standard errors are model-based in both cases.

| **Parameter** | **GLM β (SE)** | **GLMM β (SE)** |
| --- | --- | --- |
| (Intercept) | -2.367 (0.516) | -2.367 (0.516) |
| Population (Mainland Resident) | 0.584 (0.314) | 0.584 (0.314) |
| Population (Tasmanian Resident) | 0.253 (0.339) | 0.253 (0.339) |
| Life cycle complexity (Simple) | 2.119 (0.302) | 2.119 (0.302) |
| Sex (Male) | -0.056 (0.158) | -0.056 (0.158) |
| Sampling date | -0.001 (0.002) | -0.001 (0.002) |
| Life cycle complexity (Simple) × Population (Mainland Resident) | -0.701 (0.389) | -0.701 (0.389) |
| Life cycle complexity (Simple) × Population (Tasmanian Resident) | -0.974 (0.422) | -0.974 (0.422) |

**Table S10.** Pairwise Variance inflation factors (VIFs) calculated for each predictor variable considered for inclusion in the generalised linear models.

| Term | VIF |
| --- | --- |
| Model: GLM testing observed richness by sample type (blood, faecal) and host population | |
| Population | 2.10 |
| Sample Type | 3.07 |
| Sex | 1.00 |
| Sampling Date | 1.05 |
| Population x Sample Type | 2.60 |
| Model: Beta regression of local contribution to beta diversity (LCBD), blood samples | |
| Population | 1.07 |
| Sex | 1.01 |
| Sampling Date | 1.14 |
| Model: Beta regression of local contribution to beta diversity (LCBD), faecal samples | |
| Population | 1.05 |
| Sex | 1.05 |
| Sampling Date | 1.05 |
| Model: GLM testing sampling state impact on parasite community of residents and Tasmanian migrants | |
| Population | 1.06 |
| Sex | 1.06 |
| Sampling Date | 4.28 |
| State | 4.16 |
| Model: GLM testing life cycle complexity impact on parasite sharing | |
| Population | 1.06 |
| Life Cycle Complexity | 1.00 |
| Sex | 1.00 |
| Sampling Date | 1.06 |
| Model: GLM testing body condition impact on parasite community | |
| Body condition | 1.37 |
| Population | 1.22 |
| Sex | 1.04 |
| Sampling Date | 1.14 |
|  |  |
|  |  |
|  |  |

**Table S11.** Model-averaged estimates for a generalised linear model with a negative binomial error distribution predicting observed richness of parasite amplicon sequence variants for mainland residents and Tasmanian migrants sampled in New South Wales (NSW) and Queensland (QLD), treating blood and faecal samples from each bird as a single community. Coefficient estimates (β), standard errors (SE) and 95% cluster-robust confidence intervals (95% CI) are model-averaged across the two models within ΔAICc < 2; standard errors are clustered on host individual. The parameters included in each model are indicated by black dots. Full model selection details are given in Table S4.

| Parameter | 1 | 2 | β | SE | 95% CI |
| --- | --- | --- | --- | --- | --- |
| (Intercept) | • | • | -0.419 | 0.456 | (-1.312, 0.474) |
| Sampling date (day of year) |  | • | -0.001 | 0.002 | (-0.006, 0.004) |
| Host population (Mainland Resident) | • | • | 0.479 | 0.293 | (-0.096, 1.053) |
| Host population (Tasmanian Resident) | • | • | 0.185 | 0.303 | (-0.408, 0.778) |
| Sample type (Faecal) | • | • | 1.099 | 0.237 | (0.635, 1.563) |
| Host population (Mainland Resident) × Sample type (Faecal) | • | • | -0.935 | 0.357 | (-1.635, -0.234) |
| Host population (Tasmanian Resident) × Sample type (Faecal) | • | • | -1.822 | 0.381 | (-2.568, -1.076) |
| df | 7.00 | 8.00 |  |  |  |
| logLik | -401.37 | -400.84 |  |  |  |
| AICc | 817.09 | 818.13 |  |  |  |
| delta | 0.00 | 1.04 |  |  |  |
| weight | 0.63 | 0.37 |  |  |  |

**Table S12.** Beta regression model-averaged estimates for Local Contribution to Beta Diversity (LCBD) in blood and faecal samples, modelled separately because LCBD is calculated relative to its own sample set. Coefficient estimates (β), standard errors (SE) and 95% confidence intervals (95% CI) are averaged across all models within ΔAICc < 2 of the best model for each sample type (five models for blood, three for faecal). '(phi)' rows give the beta regression's dispersion parameter, modelled on the log scale and estimated in every model. The parameters included in each model are indicated by black dots. Full model selection details are given in Tables S5 and S6.

| ***Blood (n = 70)*** |  |  |  |  |  |  |  |  |
| --- | --- | --- | --- | --- | --- | --- | --- | --- |
| Parameter | 1 | 2 | 3 | 4 | 5 | β | SE | 95% CI |
| (Intercept) | • | • | • | • | • | -4.7887 | 0.1644 | (-5.1110, -4.4664) |
| Dispersion (phi), log scale | • | • | • | • | • | 7.9012 | 0.1753 | (7.5577, 8.2448) |
| Sampling date (day of year) | • | • |  |  | • | 0.0010 | 0.0009 | (-0.0008, 0.0028) |
| Sex (Male) | • |  | • |  |  | 0.0346 | 0.0497 | (-0.0628, 0.1319) |
| Host population (Mainland Resident) |  |  |  |  | • | 0.0106 | 0.0354 | (-0.0588, 0.0799) |
| Host population (Tasmanian Resident) |  |  |  |  | • | 0.0035 | 0.0238 | (-0.0430, 0.0501) |
| df | 4.00 | 3.00 | 3.00 | 2.00 | 5.00 |  |  |  |
| logLik | 340.37 | 339.18 | 338.65 | 337.37 | 340.58 |  |  |  |
| AICc | -672.12 | -672.00 | -670.94 | -670.56 | -670.23 |  |  |  |
| delta | 0.00 | 0.12 | 1.18 | 1.56 | 1.90 |  |  |  |
| weight | 0.30 | 0.28 | 0.17 | 0.14 | 0.12 |  |  |  |
| ***Faecal (n = 76)*** |  |  |  |  |  |  |  |  |
| Parameter | 1 | 2 | 3 | β | SE | 95% CI |  |  |
| (Intercept) | • | • | • | -4.6853 | 0.0397 | (-4.7632, -4.6075) |  |  |
| Dispersion (phi), log scale | • | • | • | 7.7340 | 0.1662 | (7.4083, 8.0598) |  |  |
| Sex (Male) |  | • |  | 0.0096 | 0.0305 | (-0.0501, 0.0693) |  |  |
| Host population (Mainland Resident) |  |  | • | -0.0210 | 0.0470 | (-0.1130, 0.0711) |  |  |
| Host population (Tasmanian Resident) |  |  | • | -0.0099 | 0.0373 | (-0.0829, 0.0631) |  |  |
| df | 2.00 | 3.00 | 4.00 |  |  |  |  |  |
| logLik | 365.80 | 366.10 | 367.16 |  |  |  |  |  |
| AICc | -727.43 | -725.87 | -725.75 |  |  |  |  |  |
| delta | 0.00 | 1.56 | 1.68 |  |  |  |  |  |
| weight | 0.53 | 0.24 | 0.23 |  |  |  |  |  |

**Table S13.** Support for the host population × life cycle complexity interaction across model sets defined by different ΔAICc cut-offs. The summed Akaike weight is the total weight of models within the set that contain the interaction, renormalised across that set.

| **Model set** | **Models in set** | **Models containing the interaction** | **Summed Akaike weight** |
| --- | --- | --- | --- |
| ΔAICc < 2 | 4 | 3 | 0.81 |
| ΔAICc < 4 | 7 | 4 | 0.73 |
| ΔAICc < 6 | 9 | 4 | 0.68 |
| ΔAICc < 10 | 12 | 4 | 0.66 |
| All candidate models | 20 | 4 | 0.66 |

**Table S14.** Model of scaled degrees fitted with mainland residents excluded. Estimates are from the top model according to ΔAIC <2, with cluster-robust standard errors clustered on host individual.

| **Parameter** | **β** | **SE** | **95% CI** |
| --- | --- | --- | --- |
| (Intercept) | -2.654 | 0.259 | (-3.161, -2.146) |
| Population (Tasmanian Resident) | 0.280 | 0.313 | (-0.333, 0.892) |
| Life cycle complexity (Simple) | 2.118 | 0.333 | (1.464, 2.771) |
| Life cycle complexity (Simple) × Population (Tasmanian Resident) | -0.973 | 0.418 | (-1.793, -0.153) |
| Sex (Male) | 0.083 | 0.196 | (-0.302, 0.467) |

**Table S15.** Leave-one-genus-out refits. The model was refitted omitting each simple life cycle genus in turn. The coefficient shown is the life cycle complexity (Simple) × host population (Tasmanian Resident) interaction, model-averaged across models within ΔAICc < 2 with cluster-robust standard errors.

| **Genus omitted** | **Simple genera remaining** | **Summed weight of interaction** | **β** | **95% CI** |
| --- | --- | --- | --- | --- |
| None (full data) | 3 | 0.81 | -0.973 | (-1.792, -0.155) |
| *Eimeria* | 2 | 1.00 | -1.126 | (-2.042, -0.210) |
| *Isospora* | 2 | 0.68 | -1.007 | (-1.805, -0.208) |
| *Atoxoplasma* | 2 | 0.14 | -0.853 | (-1.835, 0.130) |

**Table S16**. Model-averaged estimates for GLM predicting scaled degrees (proportion of degrees linking parasite genera to host individuals extracted from bipartite network). Coefficient estimates (β), standard errors (SE) and 95% cluster robust confidence intervals (95% CI) are based on model averaging across the top two models (ΔAICc < 2). The parameters included in each model are indicated by black dots. The intercept represents the predicted scaled degrees when all predictor variables are equal to zero or their reference (baseline) for categorical variables. For each model in the top set, we also present degrees of freedom (df), Log-likelihood (logLik), small-sample corrected Akaike Information Criterion (AICc), the difference in AICc from the top model (delta) and the Akaike weight, which reflects the relative support for each model.

| Parameter | 1 | 2 | β | SE | 95% CI | WIP |
| --- | --- | --- | --- | --- | --- | --- |
| (Intercept) | • | • | -0.993 | 0.511 | (-1.995, 0.010) | NA |
| Population (Tasmanian Migrant) | • | • | -0.213 | 0.181 | (-0.569, 0.142) | 1.00 |
| Population (Tasmanian Resident) | • | • | -0.442 | 0.169 | (-0.774, -0.111) | 1.00 |
| Sampling Date |  | • | -0.004 | 0.005 | (-0.013, 0.005) | 0.33 |
| df | 3.00 | 4.00 |  |  |  |  |
| logLik | -185.85 | -185.49 |  |  |  |  |
| AICc | 377.89 | 379.31 |  |  |  |  |
| delta | 0.00 | 1.42 |  |  |  |  |
| weight | 0.67 | 0.33 |  |  |  |  |

**Table S17.** Proportional similarity of each parasite genus compared to its own fixed-fixed null distribution, generated from 2,000 randomisations of the observed network. A genus was considered to depart from the null expectation where its observed value fell more than 1.96 standard deviations from the mean of its own null distribution. Genera detected in fewer than five individuals were excluded, because their null distributions are degenerate.

| **Genus** | **Life cycle** | **Observed PS** | **Null mean** | **95% null interval** | **z-score** |
| --- | --- | --- | --- | --- | --- |
| Leucocytozoon | complex | 0.62 | 0.88 | (0.76, 1.00) | -4.41 |
| Plasmodium | complex | 0.59 | 0.84 | (0.67, 1.00) | -2.90 |
| Lankesterella | complex | 0.41 | 0.75 | (0.51, 0.99) | -2.77 |
| Atoxoplasma | simple | 0.95 | 0.96 | (0.93, 1.00) | -0.81 |
| Isospora | simple | 0.94 | 0.94 | (0.88, 1.00) | 0.10 |
| Haemoproteus | complex | 0.94 | 0.92 | (0.84, 1.00) | 0.43 |

**References**

Bennett, G. F., Earlé, R. A. & Peirce, M. A. 1992. New species of avian *Hepatozoon* (Apicomplexa: Haemogregarinidae) and a re-description of *Hepatozoon neophrontis* (Todd & Wohlbach, 1912) Wenyon, 1926. *Systematic Parasitology,* 23, 183-193.

Box, E. D. 1981. *Isospora* as an Extraintestinal Parasite of Passerine Birds 1. *The Journal of Protozoology,* 28, 244-246.

Fayer, R. 1980. Epidemiology of protozoan infections: The coccidia. *Veterinary Parasitology,* 6, 75-103.

Keckeisen, C., Šujanová, A., Himmel, T., Matt, J., Nedorost, N., Chagas, C. R. F., Weissenböck, H. & Harl, J. 2024. *Isospora* and *Lankesterella* Parasites (Eimeriidae, Apicomplexa) of Passeriform Birds in Europe: Infection Rates, Phylogeny, and Pathogenicity. *Pathogens,* 13, 337.

Levine, N. D. 1982. The genus *Atoxoplasma* (Protozoa, Apicomplexa). *The Journal of Parasitology*, 719-723.

López-Osorio, S., Chaparro-Gutiérrez, J. J. & Gómez-Osorio, L. M. 2020. Overview of poultry *Eimeria* life cycle and host-parasite interactions. *Frontiers in Veterinary Science,* 7, 384.

Lüdecke, D., Ben-Shachar, M. S., Patil, I., Waggoner, P. & Makowski, D. 2021. performance: An R package for assessment, comparison and testing of statistical models. *Journal of Open Source Software,* 6.

Parsa, F., Bayley, S., Bell, F., Dodd, S., Morris, R., Roberts, J., Wawman, D., Clegg, S. R. & Dunn, J. C. 2023. Epidemiology of protozoan and helminthic parasites in wild passerine birds of Britain and Ireland. *Parasitology,* 150**,** 297-310.

Rapp, E. & Wolf, M. 2024. 18S rDNA sequence-structure phylogeny of the eukaryotes simultaneously inferred from sequences and their individual secondary structures. *BMC Research Notes,* 17, 124.

Valkiūnas, G. 2004. *Avian malaria parasites and other haemosporidia*, Boca Raton, FL: CRC Press.
